# Challenges in measuring individual differences in functional connectivity using fMRI: The case of healthy aging

**DOI:** 10.1002/hbm.23653

**Published:** 2017-05-23

**Authors:** Linda Geerligs, Kamen A. Tsvetanov, Cam‐CAN, Richard N. Henson

**Affiliations:** ^1^ MRC Cognition and Brain Sciences Unit Cambridge United Kingdom; ^2^ Cambridge Centre for Ageing and Neuroscience (Cam‐CAN) University of Cambridge and MRC Cognition and Brain Sciences Unit Cambridge United Kingdom; ^3^ Donders Institute for Brain, Cognition and Behaviour, Radboud University the Netherlands; ^4^ Centre for Speech, Language and the Brain, Department of Psychology University of Cambridge Cambridge United Kingdom; ^5^ Department of Clinical Neurosciences University of Cambridge Cambridge United Kingdom

**Keywords:** functional connectivity, aging, resting state, pre‐processing, functional magnetic resonance imaging, smoothing, filtering, nuisance regression, vascular health, head motion

## Abstract

Many studies report individual differences in functional connectivity, such as those related to age. However, estimates of connectivity from fMRI are confounded by other factors, such as vascular health, head motion and changes in the location of functional regions. Here, we investigate the impact of these confounds, and pre‐processing strategies that can mitigate them, using data from the Cambridge Centre for Ageing & Neuroscience (http://www.cam-can.com). This dataset contained two sessions of resting‐state fMRI from 214 adults aged 18–88. Functional connectivity between all regions was strongly related to vascular health, most likely reflecting respiratory and cardiac signals. These variations in mean connectivity limit the validity of between‐participant comparisons of connectivity estimates, and were best mitigated by regression of mean connectivity over participants. We also showed that high‐pass filtering, instead of band‐pass filtering, produced stronger and more reliable age‐effects. Head motion was correlated with gray‐matter volume in selected brain regions, and with various cognitive measures, suggesting that it has a biological (trait) component, and warning against regressing out motion over participants. Finally, we showed that the location of functional regions was more variable in older adults, which was alleviated by smoothing the data, or using a multivariate measure of connectivity. These results demonstrate that analysis choices have a dramatic impact on connectivity differences between individuals, ultimately affecting the associations found between connectivity and cognition. It is important that fMRI connectivity studies address these issues, and we suggest a number of ways to optimize analysis choices. *Hum Brain Mapp 38:4125–4156, 2017*. © **2017 Wiley Periodicals, Inc.**

## INTRODUCTION

Functional connectivity, as measured by functional magnetic resonance imaging (fMRI), has become a popular way to investigate age‐related differences in brain function and the implications of these differences for cognitive health in old age. There have been a number of consistent findings, including a reduction of connectivity within the default mode network (DMN) [Andrews‐Hanna et al., 2007; Geerligs et al., 2015a,2015b; Sambataro et al., 2010], and decreased segregation of functional networks in older adults [Betzel et al., 2014; Chan et al., 2014; Geerligs et al., 2014, 2015a, 2015b]. However, there have also been discrepancies, for example concerning the overall effect of age on functional connectivity. Many studies report both increases and decreases in functional connectivity with age [Betzel et al., 2014; Biswal et al., 2010; Chan et al., 2014; Geerligs et al., 2014, 2015a; Meier et al., 2012], whereas others report mainly age‐related decreases [Chou et al., 2013; Damoiseaux et al., 2008; Onoda et al., 2012], or age‐related increases in connectivity strength [Ferreira et al., 2016]. We suspect that these discrepancies reflect different analysis choices, designed to reduce the effects of physiological confounds. In the present study, we systematically explore a number of important confounds and the effects of different methods to address them, using two resting‐state fMRI sessions from a large sample of adults uniformly spread across the adult lifespan.

One important issue is separating the effects of age (or any other variable) on neural versus vascular components of the fMRI signal [Murphy et al., 2013; Tsvetanov et al., 2015]. Functional connectivity estimates, for example, can be confounded by physiological rhythms, such as breathing and heart rate, and different degrees of cerebrovascular reactivity [Golestani et al., 2015, 2016; Liu, 2013]. This is particularly problematic in the case of aging, given that such rhythms are known to be affected by age. Moreover, age‐related changes have been observed in the structure, vasodilatory capacity and other biomechanistic properties of cerebral blood vessels [Logothetis, 2008]. These changes could lead to disrupted autoregulation and impaired vascular reactivity [Kalaria, 2010]. Therefore, the age‐related changes observed in fMRI studies of functional connectivity may partially reflect vascular rather than true neural differences [Mark et al., 2015].

In addition to vascular effects, it has been shown that older participants tend to move more in the scanner [D'Esposito et al., 1999; Geerligs et al., 2015a,2015b]. Head‐motion during scanning has been associated with structured artefacts in fMRI timeseries. This can lead to spurious increases in connectivity between nearby voxels and decreases in connectivity between remote voxels. Even small amounts of motion during a scan can substantially affect functional connectivity estimates [Van Dijk et al., 2012; Power et al., 2012; Yan et al., 2013a]. A number of studies have proposed methods to reduce effects of motion on functional connectivity, but even after removing scans with motion artefacts and elaborate modelling of motion parameters, differences in connectivity between high and low motion participants remain [Power et al., 2014; Satterthwaite et al., 2013; Yan et al., 2013a]. This has led some studies to apply corrections at the group level, for example covarying out summary estimates of head motion for each participant. However, if head motion also changes systematically with age, such group‐level corrections may also remove true neurobiological effects of aging.

Functional connectivity analyses are also affected by the regions of interest (ROIs) used to measure connectivity. The location or number of true functional regions may change with age [Chan et al., 2014], causing misalignment with the ROIs used for analysis. This is particularly likely when ROIs are based on younger adults [Craddock et al., 2012; Gordon et al., 2016; Power et al., 2011]. Misalignment of ROIs with true functional regions will cause greater heterogeneity in the signals within ROIs, which may lead to weakened functional connectivity estimates [Geerligs et al., 2016] and seemingly reduced segregation between brain systems [Sohn et al., 2015].

In an attempt to address some of these issues, most studies use elaborate analysis pipelines. For example, studies may regress out signals from white matter (WM), cerebrospinal fluid (CSF), or global signal to reduce vascular and motion confounds. Given that respiratory and cardiac confounds may have different frequency distributions compared to true functional connectivity, band‐pass filtering of the data is also often used, while differences in the location of functional regions may be attenuated by spatial smoothing. However, the consequences of these analysis choices on the effects of aging on functional connectivity have not been explored systematically. Here, we aim to examine these analysis choices by exploiting a large dataset of resting‐state functional connectivity from over 200 individuals sampled uniformly across the adult‐lifespan from 18 to 88 years of age as part of the CamCAN project (http://www.cam-can.org). These individuals were scanned twice (a few months up to a few years apart), allowing us to look at the effect of analysis choices on within‐participant reliability.

## METHODS

### Participants

Two hundred and thirty‐six participants (18–88 years old, *M* = 53.8, standard deviation [SD] = 17.8, 119 males and 117 females) were included in this study, from the population‐based sample of the Cambridge Centre for Ageing and Neuroscience (CamCAN). The sample used in this study was identical to the sample used in a previous publication [Geerligs et al., 2016]. Participants were included if no brain abnormalities were detected and if they completed both (f)MRI testing sessions. Participants scored 25 or higher on the mini mental state exam [Folstein et al., 1975], had no contraindications to MRI, had normal or corrected‐to‐normal vision and hearing, were native English‐speakers, and had no neurological disorders [Shafto et al., 2014]. Ethical approval for the study was obtained from the Cambridgeshire 2 (now East of England ‐ Cambridge Central) Research Ethics Committee. Participants gave written informed consent.

### fMRI Data and Image Acquisition

All participants underwent two separate sessions of eyes‐closed resting state fMRI scans, which were between three months and three years apart. The time difference between the two scanning session was independent of the participants' age (*r* = +0.045, *P* = 0.52). These data were collected as part of more extensive scanning sessions in a 3T Siemens TIM Trio, with a 32 channel head‐coil. The first scan contained 261 volumes (lasting 8 min and 40 s) and the second scan contained 152 volumes (lasting 5 min). Each volume contained 32 axial slices (acquired in descending order), with slice thickness of 3.7 mm and interslice gap of 20% (for whole brain coverage including cerebellum; TR = 1,970 ms; TE = 30ms; flip angle = 78 degrees; FOV = 192 mm × 192 mm; voxel‐size = 3 mm × 3 mm × 4.44 mm). In both sessions, a high‐resolution (1 mm × 1 mm × 1 mm) T1‐weighted Magnetization Prepared RApid Gradient Echo (MPRAGE) image was acquired. In the first session, we additionally acquired a T2‐weighted structural image (1 mm × 1 mm × 1 mm) using a Sampling Perfection with Application optimized Contrasts using different flip angle Evolution (SPACE) sequence.

### Data Pre‐Processing

The data were pre‐processed using the SPM12 software (http://www.fil.ion.ucl.ac.uk/spm), as called by the automatic analysis (AA) batching system (http://imaging.mrc-cbu.cam.ac.uk/imaging/AA). The details of the pipeline can be found in Taylor et al. [2017]. In brief, the functional images were undistorted using fieldmaps and subsequently corrected for motion and different slice‐timings. The T1 and T2 images from the first session were combined to segment various tissue classes, including gray matter (GM), WM, and CSF. For the second session, the segmentation was based only on the T1 images. Next, a sample‐specific anatomical template was created for each session, based on the GM and WM segments for each participant, using the DARTEL procedure to optimize inter‐participant alignment. Each session's template was then transformed into MNI space, using a 12‐parameter affine mapping. Next, the EPI images were coregistered to the T1 image, and the DARTEL flowfields and MNI transformation applied to the EPI images. The segmented images were also used to create WM and CSF masks for each participant by selecting only voxels with less than 1% of GM and more than 80% of WM/CSF.

### Extended Pre‐Processing and ROI Extraction

We used a combination of approaches to reduce the effects of motion on the functional connectivity results. To quantify the total motion for each participant, the root mean square volume‐to‐volume displacement was computed using the approach of Jenkinson et al. [2002].The first motion‐correction step was to apply wavelet‐despiking to remove motion artefacts [Patel et al., 2014]. The method detects irregular events at different frequencies by identifying chains of outlying wavelet coefficients, and projects these out of the voxel timeseries. Because it only removes the contaminated part of the time‐frequency data, this procedure is able to retain more data than when data scrubbing is applied. The algorithm can remove different types of motion artefacts, including spin‐history effects and higher frequency events such as spikes. The spike percentage quantifies the number of corrections performed by the wavelet despiking method and represents the percentage of voxels containing a spike in each volume of data. Participants with an average spike percentage, in any of the sessions, of two SDs above the mean across both sessions (6.55%), were excluded from further analysis. This led to the exclusion of 19 participants. Four additional participants were excluded due to normalization problems, leaving a total of 214 participants included in all analyses of functional connectivity data. For analyses of individual differences in vascular health and head motion, in relation to cognitive function and GM (see below), the 19 high motion participants were not excluded, leaving a sample of 232.

**Figure 1:**
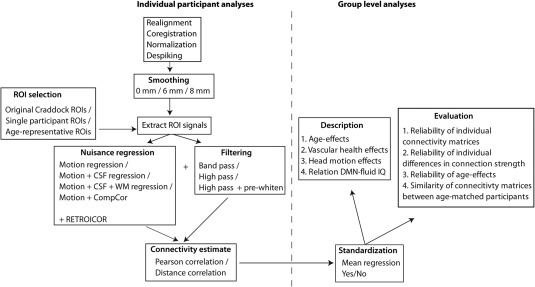
Illustration of the analysis pipelines and the various pre‐ and post‐processing steps that were compared.

In the Results section, we compared different pre‐processing pipelines. Here, we describe the default pipeline, while each section of the Results describes those aspects that were adjusted. The different pipelines are also illustrated in Figure 1; the left side of the figure corresponds to within‐participants pre‐processing steps, while the right side corresponds to between participant corrections and analyses.

After wavelet despiking, the data were smoothed with an 8 mm full‐width half maximum (FWHM) kernel and signals were extracted from 748 of the 840 regions defined by Craddock et al. [2012]. Only 748 regions were included because they had sufficient coverage in our recent paper using a superset of the participants included here [Geerligs et al., 2015a, 2015b], which allowed us to use existing network labels defined in our previous study. We excluded two more ROIs with insufficient coverage in the second session, resulting in a set of 746 remained ROIs.

The second step to reduce the effects of motion and other noise confounds on functional connectivity results was to apply a general linear model (GLM). This model included expansions of the six original motion parameters, as well as of average signals in the WM and CSF from the time courses of each voxel within each ROI. The WM and CSF signals were created by averaging across voxels in the associated mask image, after the wavelet‐despiking but before smoothing. The expansions included the first‐order temporal derivative, as well as their squares and squared derivatives, which reduces the effects of motion [Satterthwaite et al., 2013]. In total, there were 32 confound and noise regressors. A high‐pass filter (0.008 Hz) or band‐pass filter (0.008–0.1 Hz) was implemented by including a discrete cosine transform set in the GLM, ensuring that nuisance regression and filtering were performed simultaneously. Unless mentioned otherwise, analyses reported in the results section are based on pre‐whitened, high‐pass filtered data. The autocorrelation in the GLM error was modelled by a family of 8 exponentials with half‐lives from 0.5 to 64 TRs, given evidence that an AR(1) plus white noise model is not sufficient for resting‐state data [Eklund et al., 2012]. The autocorrelation hyperparameters were estimated using Restricted Maximum Likelihood Estimation. Efficiency of estimating the autocorrelation hyperparameters was increased by pooling across voxels within each ROI, but done separately for each ROI, to allow for true differences in autocorrelation between functional regions. The autocorrelation model was inverted to pre‐whiten the data [Friston et al., 2002] and functional connectivity was then estimated from the whitened residuals of this model.

In our analyses, we found evidence for residual physiological effects on connectivity estimates, after the default pre‐processing steps described above. Therefore, we investigated whether using the CompCor method [Behzadi et al., 2007] could reduce these effects. To this end, we extracted data from the WM and CSF masks described above (Data Pre‐Processing section) after the data were wavelet‐despiked, but before smoothing. Principal component analysis was applied to combined voxels from the CSF and WM data and the first five principal components were retained and regressed out of the functional data [Chai et al., 2012]. Thus, in the CompCor analyses, we used the five combined CSF and WM components, instead of the mean CSF and WM signals with its derivatives and squared terms (8 in total) that we used in the default processing pipeline.

We compared two different methods of computing functional connectivity. One was the traditional Pearson correlation (Pcor), which was computed based on the average signal across all voxels within each ROI. The other was a multivariate method, called distance correlation (Dcor) [Székely et al., 2007]. The most important differences between Pcor and Dcor are that (1) Dcor can measure both linear and nonlinear associations between regions, while Pcor only detects linear associations, (2) Pcor can distinguish between positive and negative correlations while Dcor cannot; it only measures the strength of the association between two regions, and (3) Dcor is a multivariate measure of functional connectivity, while Pcor can only measure univariate associations. That is why Dcor was computed based on all the individual voxel time‐series within each ROI (without averaging first). The details of the Dcor computation can be found in our recent paper [Geerligs et al., 2016]. We used the unbiased version of Dcor, which is not affected by the number of voxels in an ROI [Székely and Rizzo, 2013].

Previous work has shown that standardization methods, which correct individual differences in mean connectivity, may be necessary to minimize the influence of nuisance variables on inter‐individual variation in functional connectivity [Yan et al., 2013b]. Here, we assessed the effect of mean regression (MR) in the context of aging, given that MR has been shown to be highly effective without introducing artefactual differences between participants [Yan et al., 2013b]. After connectivity estimates were obtained for each participant, the mean connectivity value over all connections for each participant was regressed out of each connection. This was done using a regression model with an intercept term, which modelled the average connectivity across all participants. Therefore, to compute the intra‐class correlation (ICC) accurately, the mean connectivity across participants was added back onto the connectivity estimates of each participant after mean connectivity had been regressed out. While MR corrects for within‐session effects in mean connectivity, it does not address between‐session differences in the mean across participants. To remove differences in the mean connectivity between sessions (across ROIs and participants), we replaced the grand mean of the connectivity estimates of each session by the mean of connectivity estimates across both sessions.

### Cognitive Measures

Participants performed several cognitive tasks outside the scanner as part of a larger test battery (for a full description) [see Shafto et al., 2014]. Here, we focus on measures of general cognitive function. Fluid intelligence was measured by the Cattell Culture Fair, a timed pen and‐paper test in which participants perform a series of nonverbal puzzles [Cattell and Cattell, 1960]. Crystallized intelligence was measured using the Spot‐the‐Word Test [Baddeley et al., 1993], in which participants see pairs of words and non‐words pairs and decide which is the real word. Verbal memory was measured using delayed recall performance on the logical memory test from the Wechsler Memory Scale Third UK edition, in which participants listen to two brief stories and recall them 25–30 min later (WMS‐III UK) [Wechsler, 1999]. Finally, on the speeded choice response time (RT) task, participants used a 4‐button response box and responded as quickly as possible (maximum 3s) to 1 of 4 possible cued fingers (67 trials, variable inter‐trial interval with a mean of 3.7 s). Incorrect trials (2.8% on average) and outlier RTs that were >3 SDs away from an individual's mean were removed (1.3% of correct trials on average). The mean (M‐RT) and the RT variability (SD of RT values, SD‐RT) were computed from remaining trials, and inverted (*y* = *x* ^−1^) to obtain a more Gaussian distribution across participants. Due to this inversion, higher scores are related to better performance for each of the cognitive measures. Data from 12 participants were missing for the choice RT task because of equipment error and 2 participants were excluded because they made too many errors (less than 50% correct, final *N* = 218).

### Vascular Health and Pulse‐Oximetry Data

We measured vascular health based on an electrocardiogram (ECG) collected during a separate magnetoencephalography (MEG) scanning session. The sampling frequency for the ECG data was 1 kHz. To detect the ECG R‐peaks corresponding to each heartbeat, we used the PeakFinder function in MatlabCentral and calculated the interbeat interval (IBI) as the time difference between each pair of subsequent R‐peaks. Next, we derived measures of mean heart rate and low (LF‐HRV) and high frequency (HF‐HRV) heart rate variability (0.05–0.15 Hz; LF‐HRV and 0.15–0.4 Hz; HF‐HRV) using HRV Analysis Software [Ramshur, 2010]. The details of this procedure, including methods of outlier detection are described in Tsvetanov et al. [2015]. LF and HF‐HRV were log‐transformed to obtain more normal distributions. Next, we applied principal component analysis and used the first principal component of these measures as a summary to study age differences in vascular health [Varadhan et al., 2009].Vascular health measures were not present for 36 participants in session 1 and 30 participants in session 2. In addition, participants with outlying scores on one of the measures (after removing main effects of age) were excluded (8 participants in session 1 and 3 participants in session 2).

Additional physiological data were acquired during our fMRI scans using a pulseoximeter with sampling frequency 50 Hz, placed on the left index finger of the participant. Preprocessing of the pulseoximetry data was performed in Matlab (MATLAB 8.1, The MathWorks, Natick, MA, 2013) using the Tapas PhysIO Toolbox (http://www.tnu-zurich.org/tapas), where detected IBIs were downsampled to EPI sampling rate (TR = 1.97, at the first slice, as reference, of each scan volume). High quality pulseoxymetry data, in which the heartbeats could be detected clearly, was available for 138 participants. The pulseoxymetry data was used to create six physiological noise regressors (RETROICOR) using a well‐established model‐based approach (sine and cosine Fourier phase expansions of the heart beat and an additional regressor for IBI outliers) [Glover et al., 2000]. In one of the analyses options in the Results section, these physiological noise regressors were used as covariates of no interest in addition to the other covariates such as motion parameters and CSF + WM signals.

### Voxel‐Based Morphometry

Voxel‐based morphometry (VBM) was used to estimate GM volume at each voxel [Ashburner and Friston, 2000]. The segmented GM images were modulated by Jacobian determinants to adjust for volume changes during the DARTEL‐MNI transformations, and smoothed with a 10 mm FWHM Gaussian kernel. Multiple regression was used to relate GM at each voxel to trait motion or vascular health. Trait motion was defined as the average motion across both fMRI scanning sessions. Similarly, for this analysis “trait” vascular health was defined as the average vascular health across both sessions. A linear and a quadratic age term, as well as gender were included as covariates of no interest. In addition, total intracranial volume (TIV) was included as a nuisance regressor in the analyses of vascular health effects, to reduce effects of head size, while total gray matter volume (TGM) was included in the analyses of trait motion effects, to additionally reduce the impact of motion artefacts [Reuter et al., 2015]. Analyses were restricted to a mask created by determining the threshold at which a binarized GM image was maximally correlated with the average GM image [Ridgway et al., 2009]. To correct for multiple comparisons across voxels, statistical maps were thresholded at *P* < 0.001, and clusters of suprathreshold voxels identified that survived *P* < 0.05 family‐wise error corrected for their extent using random field theory.

### Parcellations

For each participant, we constructed parcellations based on their fMRI data using the pipeline developed by Craddock et al. [2012]. To make our data compatible with this pipeline, we applied a very similar pre‐processing pipeline prior to creating the parcellations. We applied the same amount of smoothing (6‐mm Gaussian kernel) and we regressed out CSF and WM signals and applied a band‐pass filter (for details on implementation, see section on extended pre‐processing and ROI extraction). The residuals of this analysis were saved and used as input for the parcellation algorithm. Next, the distances between voxels were determined by correlating the time course of each voxel and that of its 26 nearest neighbors. A threshold of *r* > 0.5 was applied to correlation coefficients to exclude negative and weak correlations. Then the normalized cut spectral clustering (NCUT) algorithm was applied to identify clusters for each participant and each session. To make the parcellation comparable to the original Craddock parcellation which we have used in the remainder of this article, we pre‐specified the number of clusters as 750 and restricted the voxels to those covered by the ROIs that we used from the original set. It should be noted that the NCUT algorithm can result in empty clusters, leading to a number of ROIs that is less than 750. For each participant, we obtained a final clustering solution by combining the parcellations from the two sessions in a group clustering. In this group level clustering, the distances between voxels were determined by their cluster membership. Next, we also obtained an age‐representative parcellation by combining each participant's final parcellations in another group level clustering analysis. The similarity between different parcellations was assessed using normalized mutual information (NMI). NMI measures how much information is provided by one set of assignments about another set of assignments [Strehl and Ghosh, 2003] and varies from 0 (no mutual information) to 1 (identical node assignments).

### Evaluation of the Connectivity Measures

We used different approaches to compare the connectivity estimates for the different pre‐ and post‐processing options. All analyses were performed on data from session 1, except from analyses of reliability of connectivity.

First, we investigated the strength and distribution of associations between age and functional connectivity, as well as the association between head motion and functional connectivity, and vascular health and functional connectivity. To test whether differences between processing options were significant, we used permutation tests with 5,000 random assignments. In each permutation run, we randomly allocated the results of each ROI‐pair (containing correlations between age and connectivity, or between vascular health and connectivity) to one of two groups. Then, the mean effect (of head motion or vascular health) was computed within each of these groups, and the difference between these groups compared to the real difference we measured between the processing options. The *P*‐value was determined by looking at the proportion of times that the absolute value of the random difference was larger than the absolute value of the real difference between options.

Second, we computed three complementary indices of reliability. One index was the ICC between the (vectorized) connectivity matrices of the two sessions for each participant (within‐participant reliability). In this way, we tested whether the absolute values and the regional differences in connectivity strength remained stable across sessions. This reliability measure was compared across processing options using the Wilcoxon signed rank test. Another method focused on the stability of individual differences, by comparing individual differences in connectivity strength across sessions using the ICC, separately for each pair of ROIs (between‐participant reliability). We then averaged these ROI ICC values across all within‐network and all between‐network connections. For this measure of reliability, we again used permutation tests to determine the significance of the differences (similar to the effects of age and vascular health above). The final reliability measure concerned the effect of age, by comparing the (vectorized) matrices of correlations between age and connectivity across sessions using the ICC. In this way, we tested whether the absolute values and the regional differences in the association between age and connectivity remained stable across sessions. For this measure, we computed the significance of the differences using a slightly different permutation test. We started with estimates of the association between age and connectivity for two sessions and two processing options. Next, we randomly shuffled age‐connectivity associations across processing options. Importantly, the same shuffle was performed for both sessions. Next, we computed the reliability of the age‐effect in both reshuffled datasets and we compared the difference in reliability between the two sets to the difference in reliability we observed in the unshuffled data. The *P*‐value was determined by looking at the proportion of times that the absolute value of the random difference was larger than the absolute value of the real difference between processing options.

The third approach was to compute the similarity of connectivity estimates between age‐matched participants. For each participant, we found the 30 participants that most closely matched their age. We computed the similarity between the connectivity of this participant and the average connectivity of the age matched participants, using Pearson correlations. We used Pearson correlations instead of the ICC because we did not expect perfect correspondence between absolute connectivity values across participants, but we did expect a similar network structure. Here, we used the Wilcoxon signed rank test to determine the significance of the differences across processing options.

We also examined whether different pre‐and post‐processing choices affected how well connectivity measures could predict individual differences in cognitive function. Here, we focused specifically on the Cattell task of fluid intelligence as a general index of age‐related cognitive decline. For our connectivity measure, we looked at the average level of connectivity within the DMN because it has been consistently shown to decline with age, as well as mild cognitive impairment and Alzheimers's disease, and to be related to cognitive functioning [Hafkemeijer et al., 2012; Wang et al., 2013]. A test to compare overlapping correlation coefficients was used to compute the significance of the differences [Steiger, 1980].

Other analyses were specific to the question of interest in each section and the measures we used in each section are explained there. Results are reported with two decimals precision throughout the article, except when three decimals were needed to demonstrate significant differences.

## RESULTS

A summary of the analysis choices explored is shown in Table 1. We started by relating functional connectivity estimates to vascular health, as estimated from independent data.

**Table 1:** Description of the different pre‐ and post‐processing options evaluated in the different paragraphs of the Results section

Results section	Nuisance regression	Filter	Smooth	Standardize	Connectivity method
The Effects of Vascular Health and Head Motion section	CW	HP‐PW	8 mm	—	Pcor
Accounting for Remaining Physiological and Motion Signals section	N/C/CW/CC	HP‐PW	8 mm	MR/no MR	Pcor
Filtering and Autocorrelation section	CW	BP/HP/HP‐PW	8 mm	MR	Pcor
Functional Regions and ROI Homogeneity section	CW	HP‐PW	0/6/8 mm	MR	Pcor/Dcor
**Recommendation**					
Option 1	CW	HP‐PW/BP	8 mm	MR	Pcor
Option 2	CW	HP‐PW	0 mm	MR	Dcor

The final rows show the recommended pre‐ and post‐processing steps.

*Abbreviations*: BP = band pass filter; C = motion regressors + CSF regressors; CC = motion regressors +CompCor CSF and WM regressors; CW= motion regressors + CSF regressors + white matter regressors; Dcor = Distance correlation; HP = high pass filter; MR = mean regression; N = only motion regressors; Pcor = Pearson correlation; PW = pre‐whitening.

### The Effects of Vascular Health and Head Motion

**Figure 2:**
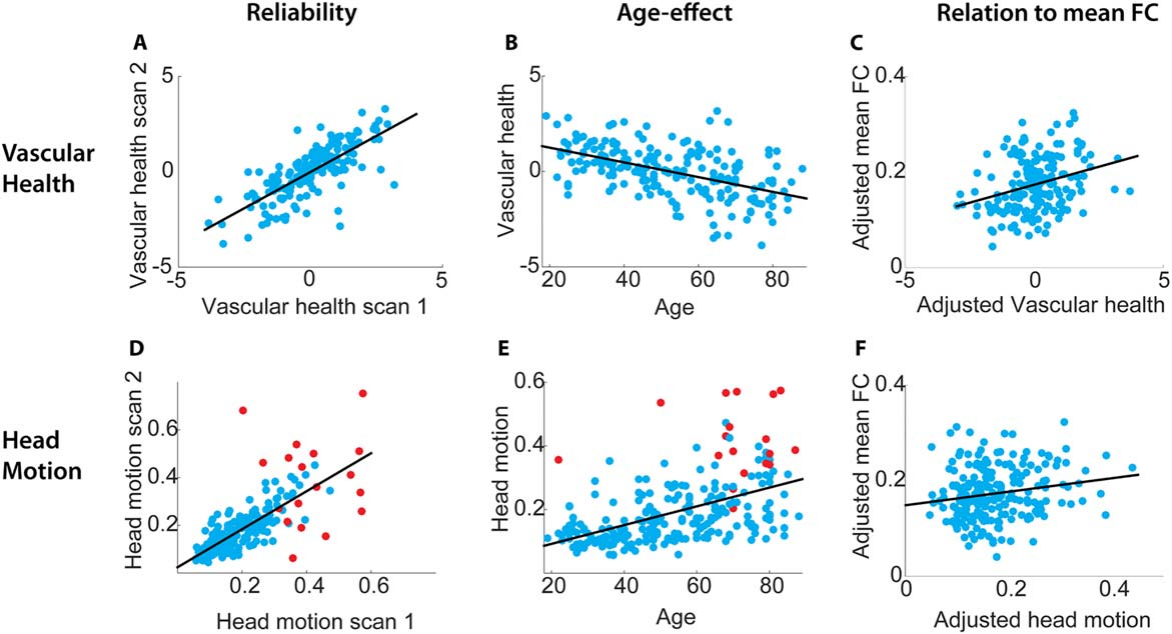
Scatterplots of the associations between (**A**) age and vascular health (scan 1), (**B**) vascular health in scans 1 and 2 (reliability), (**C**) vascular health and mean functional connectivity (FC), adjusted for effect of age, (**D**) age and head motion (scan 1), (**E**) head motion in scans 1 and 2, (**F**) head motion and mean FC, adjusted for effect of age. Red dots in the plots with head motion indicate the participants who were excluded from the functional connectivity analyses due to the high number of spikes removed by wavelet despiking.

**Figure 3:**
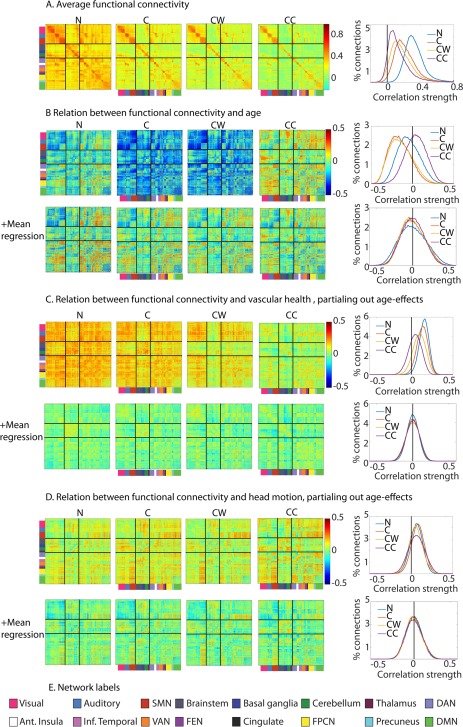
Comparison of different nuisance regression options. (**A**) Average functional connectivity across participants ROIs are ordered by functional network, as indicated by the colors on the left side and bottom of the functional connectivity matrices, based on Geerligs et al. [2015b]. The network labels are shown below, in panel E. The solid black lines differentiate sensorimotor networks (top), subcortical networks (middle), and higher cortical networks (top). The histograms show the distributions of the effects in the figures on the left. (**B**) Relation between age and functional connectivity. (**C**) Relation between vascular health and functional connectivity, after adjusting for effects of age. (**D**) Relation between head motion and functional connectivity, after adjusting for effects of age. (**E**) Network labels. SMN = somato‐motor network (SMN), DAN = dorsal attention network, VAN = ventral attention network, FEN = fronto‐executive network, FPCN = fronto‐parietal control network, DMN= default mode network, Ant = anterior, Inf.=inferior. N = only motion regressors; C = motion regressors + CSF regressors; CW= motion regressors + CSF regressors + white matter regressors; CC = motion regressors +CompCor CSF and WM regressors.

**Figure 4:**
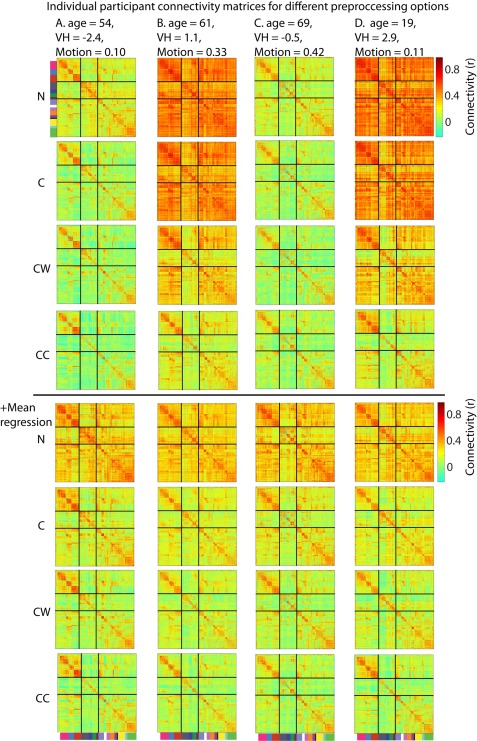
(**A**–**D**) Connectivity estimates across pre‐processing options for individual participants who vary in their age, vascular health and head motion. Participants B and D have relatively high vascular health estimates, while participants A and C have relatively low vascular health. Participants A and D have relatively high levels of head motion, while participants B and C have low levels of head motion. N = only motion regressors; C = motion regressors + CSF regressors; CW= motion regressors + CSF regressors + white matter regressors; CC = motion regressors +CompCor CSF and WM regressors.

**Figure 5:**
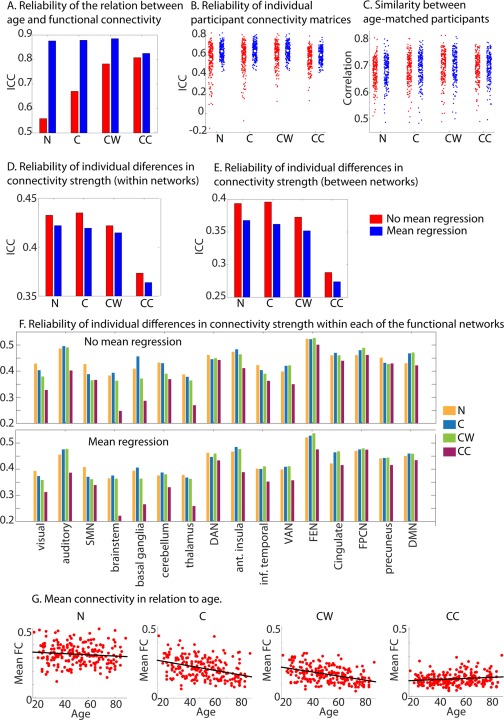
Reliability and between participant similarity for the various methods used to correct for physiological noise. (**A**) Reliability of the age‐connectivity matrices. (**B**) Reliability of each participants connectivity matrix. (**C**) Similarity between age‐matched participants. (**D**) Reliability of individual differences in connectivity strength, averaged across all within network connections. (**E**) Reliability of individual differences in connectivity strength, averaged across all between network connections. (**F**) Reliability of individual differences in connectivity strength averaged across all connections within each of the functional networks. (**G**) Scatterplot of individual differences in mean connectivity in relation to age for different pre‐processing steps. This was not shown for the mean regression steps, because mean regression removes all variations in mean connectivity. N = only motion regressors; C = motion regressors + CSF regressors; CW= motion regressors + CSF regressors + white matter regressors; CC = motion regressors +CompCor CSF and WM regressors. SMN = somato‐motor network (SMN), DAN = dorsal attention network, VAN = ventral attention network, FEN = fronto‐executive network, FPCN = fronto‐parietal control network, DMN= default mode network, Ant = anterior, Inf.=inferior.

First, we looked at the impact of vascular health and head motion on connectivity estimates after the typical nuisance regressions techniques were applied, including regression of motion parameters, CSF and WM signals and their derivate and quadratic terms. We estimated vascular health from ECG data collected during separate MEG scans [Tsvetanov et al., 2015]. This estimate of vascular health was highly reliable across sessions (Fig. 2A; *r* = 0.75, *P* < 0.001). This measure declined with age (Fig. 2B; *r* = −0.50, *P* < 0.001), paralleling the decline in mean functional connectivity (Figs. 2C and 5G), when averaged across ROI pairs (*r* = −0.43, *P* < 0.001). Moreover, participants with better vascular health had higher connectivity estimates in cortical networks (e.g., within and between sensorimotor and higher‐order networks), even after accounting for the (linear) effects of age (see Fig. 3C, heading CW). These results suggest that age‐related differences in vascular health explain some of the age‐related differences in average functional connectivity strength in cortical regions. Indeed a mediation analysis showed that vascular health significantly mediated the association between age and average functional connectivity (*a* = −0.51, *b* = 0.32, *ab* = −0.17, *t* = −3.65, *P* < 0.001, reduction in c‐path, 40%). The lack of cortical specificity of these effects suggests that vascular health is associated with transiently varying global physiological signals, related to fluctuations in breathing or blood flow for example [see also Power et al., 2017]. This is also illustrated in Figure 4A–D (heading CW) where connectivity matrices are shown for individuals with high and low levels of vascular health.

Total head motion during the scan was estimated based on the motion parameters. Head motion estimates were highly reliable across scans (Fig. 2D; *r* = +0.77, *P* < 0.001) and strongly positively correlated with age (Fig. 2E; *r* = +0.50, *P* < 0.001). After partialing out effects of age, head motion was positively related to mean connectivity (Fig. 2F; *r* = +0.18, *P* = 0.007) and the effects of head motion on functional connectivity were more localized than effects of vascular health (see Fig. 3D, heading CW). In particular, higher levels of head motion were associated with stronger connectivity between the somatomotor network (SMN in Fig. 3) and higher cortical areas, and between various higher‐order networks.

### Accounting for Remaining Physiological and Motion Signals

These associations between vascular health, head motion and functional connectivity occurred despite our elaborate pre‐processing pipeline that included regression of signals from the WM and CSF to remove non‐neural aspects of the signal. To examine the effectiveness of different pre‐processing steps, we examined how the association between age, head motion, vascular health and functional connectivity varied according to type of nuisance signal regression. We compared four different options: no nuisance signals (except for motion parameters and their derivatives, N), CSF signal regression (C), CSF + WM signal regression (CW), and CompCor (CC). For the CompCor method [Behzadi et al., 2007], we included the first 5 principal components of the CSF and WM signals as nuisance regressors. We also investigated the effects of a standardization technique called MR, in which the mean connectivity across connections of each participant is regressed out of each connectivity estimate. This has previously been advocated as a promising technique to correct for differences in mean connectivity as well as effects of head motion [Saad et al., 2013; Yan et al., 2013b]. This approach assumes that we are most interested in the effects of age on the pattern of connectivity across ROIs (i.e., is blind to any age‐related differences in mean connectivity).

Across all participants, we observed that regressing out nuisance signals led to sparser connectivity matrices, with reduced number of between network connections (Fig. 3A). Moreover, we found that nuisance regression reduced the association between vascular health and functional connectivity (Fig. 3C), confirming our hypothesis that the association is due to transiently varying global physiological signals. To summarize the association, we report the mean of the absolute values of the correlations between all ROIs in Table 2. The correlations across ROIs between vascular health and connectivity (after accounting for effects of age) were most pronounced when only motion regressors were used and were reduced significantly with each additional nuisance signal (C to CW and CW to CC). An even more pronounced reduction of vascular health effects was observed when MR was applied; this was true across all four nuisance regression options. After MR, the differences between nuisance regression options in the effects of vascular health on connectivity were minimal, although N and CC showed smaller residual effects than C and CW.

**Table 2:** Summary of main outcome measures for different nuisance and mean regression options

		N	C	CW	CC
RA	No MR	0.56	0.67	0.78	0.81
	MR	0.88	0.88	0.89	0.83
RP	No MR	0.56	0.58	0.61	0.57
	MR	0.64	0.64	0.65	0.60
SP	No MR	0.679	0.689	0.699	0.694
	MR	0.683	0.691	0.700	0.697
RSW	No MR	0.433	0.435	0.422	0.373
	MR	0.422	0.419	0.415	0.364
RSB	No MR	0.394	0.396	0.373	0.288
	MR	0.368	0.362	0.352	0.274
VH	No MR	0.18	0.16	0.12	0.09
	MR	0.07	0.07	0.08	0.07
HM	No MR	0.10	0.11	0.10	0.11
	MR	0.09	0.09	0.09	0.09

*Abbreviations*: C = motion regressors + CSF regressors; CC = motion regressors +CompCor CSF and WM regressors;. CW= motion regressors + CSF regressors + white matter regressors; HM= Average (absolute) association between head motion and connectivity; MR = mean regression; N = only motion regressors; RA = Reliability of the age‐effects; RP= Reliability of each participant's connectivity matrix; RSB= Reliability of single between‐network connections; RSW= Reliability of single within‐network connections; SP= Similarity between participants in the same age‐range; VH= Average (absolute) association between vascular health and connectivity.

With regard to the effects of head motion, we again found a significant advantage of using between‐participant MR, regardless of the within‐participant nuisance regression option that was used (see Fig. 3D and Tables 2 and 3). The differences between nuisance regression options were not very clear, either with or without MR; the associations between head motion and connectivity were most prominent for C and CC when MR was not used, while only minimal differences were observed when MR was used. In addition, the associations between connectivity and motion became more regionally‐specific as more nuisance regressors were added, with the most pronounced connectivity increases between the motor cortex and higher‐order cortical regions.

**Table 3:** Statistical tests of main outcome measure for different nuisance and mean regression options

		noMR C vs. N	noMR CW vs. C	noMR CC vs. CW	N MR vs. noMR	C MR vs. noMR	CW MR vs. noMR	CC MR vs. noMR
RA	Diff	0.11	0.11	0.03	0.32	0.21	0.1	0.02
	Pval	<0.001	<0.001	<0.001	<0.001	<0.001	<0.001	<0.001
RP	Zval	4.86	4.36	−6.58	11.11	10.12	9.03	10.05
	Pval	<0.001	<0.001	<0.001	<0.001	<0.001	<0.001	<0.001
SP	Zval	6.94	8.73	−2.24	3.24	1.80	−0.02	4.86
	Pval	<0.001	<0.001	0.025	0.001	0.072	0.984	<0.001
RSW	Diff	0.002	−0.013	−0.049	−0.011	−0.016	−0.007	−0.010
	Pval	<0.001	<0.001	<0.001	<0.001	<0.001	<0.001	<0.001
RSB	Diff	0.002	−0.023	−0.085	−0.026	−0.034	−0.021	−0.014
	Pval	<0.001	<0.001	<0.001	<0.001	<0.001	<0.001	<0.001
VH	Diff	−0.02	−0.04	−0.04	−0.12	−0.09	−0.05	−0.01
	Pval	<0.001	<0.001	<0.001	<0.001	<0.001	<0.001	<0.001
HM	Diff	0.02	−0.02	0.02	−0.01	−0.02	−0.01	−0.02
	Pval	<0.001	<0.001	<0.001	<0.001	<0.001	<0.001	<0.001

*Abbreviations*: C = motion regressors + CSF regressors; CC = motion regressors +CompCor CSF and WM regressors; CW= motion regressors + CSF regressors + white matter regressors; Diff = difference between the two estimates, this is reported for comparisons where permutation test are used; HM = Average (absolute) association between head motion and connectivity; MR = mean regression; N = only motion regressors; noMR = no mean regression; Pval = *P*‐value; RA = Reliability of the age‐effects; RP = Reliability of each participant's connectivity matrix; RSB = Reliability of single between‐network connections; RSW = Reliability of single within‐network connections; SP = Similarity between participants in the same age‐range; VH = Average (absolute) association between vascular health and connectivity; Zval = *z*‐value from the Wilcoxon signed rank test.

Importantly, we found a striking effect of nuisance regression on the association between age and functional connectivity (Fig. 3B). When no nuisance signals were regressed out, 12% of connections were significantly increased with age, while 33% of connections decreased. However, after CSF regression, the distribution of the age‐effects shifted toward lower values, and now 64% of connections showed a significant decrease with age and only 3% of connections showed an increase. The results were very similar when WM signal regression was added (60% negative, 4% positive). In contrast, when CC was performed, more positive (29%) than negative (12%) associations between age and connectivity were observed (see Fig. 3B). These changes paralleled the changes in the association between age and mean connectivity with different nuisance regression options (see Fig. 5G). MR resulted in more balanced effects of age, with approximately equal numbers of negative and positive associations. In the case of MR, the number of significant associations between age and connectivity decreased as more nuisance regressors were added (N, pos = 24%, neg = 25%; C, pos = 21%, neg = 22%; CW, pos = 20%, neg = 21%; CC, pos = 19%, neg = 20%).

To determine whether this effect of nuisance regressors was only a shift in the distribution of age‐effects, rather than a change in the pattern of age‐related changes, we used Pearson correlations to compare the matrices of age‐effects in Figure 3B. The pattern of age‐related changes was very similar for N, C, and CW, while it showed a more pronounced change for CC (N‐C, *r* = 0.91; C‐CW, *r* = 0.96; CW‐CC, *r* = 0.81). These results are encouraging, as they suggest that the changes after different pre‐processing steps (at least C and CW) primarily affect the mean of connectivity estimates, while the pattern of age effects is less affected. This is supported by the correlations between results with and without MR, which were very high for all nuisance regression options (N, *r* = 0.99; C, *r* = 0.95; CW, *r* = 0.94; CC, *r* = 0.99).

To demonstrate how connectivity estimates of individual participants were affected by these different nuisance regression and MR steps, we show the connectivity matrices of four representative participants in Figure 4. These participants varied in their age, vascular health (high for B and D; low for A and C) and head motion (high for A and D, low for B and C). Figure 5G additionally shows the variations in mean connectivity across participants for the different nuisance regression steps. The figures support and clarify some of the results we reported previously. First, we can see that without any nuisance regressors, there are extensive differences between participants, due to differences in mean connectivity between ROIs. The size and lack of specificity of these effects suggest that these do not have a neural origin, but are due to differences in physiological signals. These differences in mean connectivity are reduced but not abolished as more nuisance regressors are added. When mean differences are not corrected adequately, connectivity differences between participants cannot be estimated in a valid manner. MR deals with these differences in mean connectivity and results in comparable connectivity matrices across participants. Second, adding additional nuisance regressors increases the specificity of the connectivity matrices (e.g., stronger within and weaker between network connections). However, these changes are mainly driven by a subset of participants who showed strong connectivity between all ROIs prior to nuisance signal regression. Other participants already showed clear differentiation of within and between network connections prior to any additional nuisance regression. Finally, Figure 5G additionally shows that variations in mean connectivity are not only present in aging samples; in fact a large amount of variation is present in the younger participants as well.

We also investigated complementary indices of reliability for these different nuisance regression options. First, we looked at the reliability of the complete connectivity matrix for each participant using the intraclass correlation coefficient (ICC). This analysis asks how similar the connectivity matrices for each participant are at timepoints 1 and 2. The reliability of connectivity matrices of individual participants improved from N to C and CW. However, a significant decrease was observed from CW to CC (see Fig. 5B). When MR was performed we found a significant increase in reliability across all four nuisance regression options. The highest reliability was found for CW + MR. Next, we investigated the reliability across sessions of individual differences in connectivity strength, using the ICC. This was done for each connection separately and subsequently averaged across all within and between network connections. We observed that this form of reliability did not improve with additional nuisance regression steps. Both within and between‐network connections showed only minimal differences in reliability between N, C, and CW. However, for CC a significant decrease in reliability was observed. We also observed a decline in reliability for MR across all four conditions; however, this decline was far less pronounced than the decline we observed for CC compared to CW (see Fig. 5D,E). To further investigate these changes in the reliability of single connections, we also computed the average reliability within each of the 16 functional networks (see Fig. 5F). We found that the observed drop in reliability between CW and CC was mainly due to ROIs in subcortical regions; for example, the thalamus, basal ganglia and brainstem. However, less pronounced declines were also observed in other networks, such as the auditory and ventral attention networks. Across all processing options, there were consistent differences between functional networks in the reliability of single connections. Higher‐order cortical networks generally had more reliable connectivity values than other networks, particularly subcortical networks.

We also investigated the reliability of the age‐effect, by looking at the similarity of the age‐connectivity correlation matrices from both sessions. Interestingly, we observed that the reliability of the age‐effect behaved in line with the reliability of the individual connectivity matrices; it increased as more nuisance regression steps were added from N, to C, to CW. In contrast to the results for individual connectivity matrices, we also observed an increase for CC compared to CW. When MR was performed, the age‐effects were substantially more reliable for N, C, and CW, but less reliable for CC. Overall CW + MR was associated with the most reliable age effects. Finally, we examined the similarity between age‐matched participants (30 participants with most similar age). This index of reliability also increased significantly as more nuisance regression steps were added, and was also significantly higher when MR was performed, but significant differences were only observed for N and CC (see Fig. 5C and Table 3).

A more sophisticated method to remove physiological artefacts is RETROICOR [Glover et al., 2000], which utilizes physiological timeseries acquired during scanning. In a sub‐sample of 138 participants, we had pulse oximetry data of sufficient quality to perform RETROICOR. The association between vascular health and connectivity was smaller in this subsample of participants (CW: *M* = 0.116). After correcting for WM and CSF signals, RETROICOR resulted in a significant, although small, reduction in the association between vascular health and functional connectivity (CW + RETROICOR: *M* = 0.111, *P* < 0.001).

These results suggest that regression of CSF and WM signals can substantially attenuate the effects of vascular health on functional connectivity, while RETROICOR provides only a small further attenuation. CompCor did result in a significant further attenuation of the effects of vascular health on functional connectivity, but at the expense of a decrease in reliability. In contrast, MR resulted in increased performance for most reliability indices, as well as reduced associations between connectivity, head motion, and vascular health. Overall, we found that the combination of CW and MR gave the best performance, with high reliability estimates and relatively low effects of vascular health and head motion on connectivity estimates. In the remainder of the article, we used connectivity estimates in which CSF and WM regression, as well as MR had been applied (i.e., CW + MR). We also observed that nuisance regression shifts the distribution of age effects to lower values (except for CompCor), but has little effect on the pattern of age effects across connections. It has been suggested previously that reliability of individual connections is most important for the reliability of group‐effects on connectivity [Varikuti et al., 2017]. However, we observed that improved reliability of the age effect was associated with the reliability of each participant's full connectivity matrix (within‐participant reliability), but not with the reliability of the individual differences in connectivity estimates in each ROI pair (between‐participant reliability).

### Sensitivity of Specific Connections to Nuisance Variables

**Figure 6:**
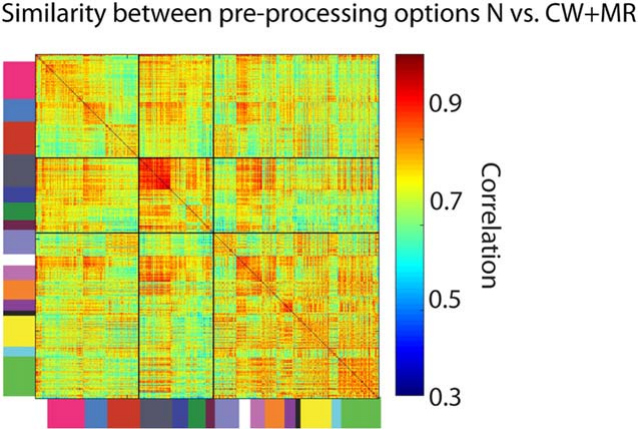
Sensitivity of the various within and between network connectivity estimates to changes in nuisance regression procedures. This figures shows for each ROI pair, the correlation across participants between the connectivity estimates of two different pre‐processing pipelines; the N (motion regressors only) connectivity estimates, and the CW + MR connectivity estimates (motion regressors +CSF and white matter regressors + mean regression).

In the previous section, we have shown that the combination of CSF and WM nuisance signals and MR reduces the associations between vascular health, head motion, and functional connectivity, and generally leads to more reliable connectivity estimates. Here, we investigated which of the connections within‐ and between‐networks were most sensitive to changes in the nuisance regression procedures. These connections may also be the ones that are most vulnerable to confounds. To investigate this, we computed the correlation between the connectivity estimates before and after nuisance regression (i.e., N vs. CW + MR). This shows the extent to which differences in connectivity estimates between participants remained the same for these different nuisance regression options. We observed particularly low correlation values for connections between subcortical (basal ganglia, thalamus, and cerebellar) and higher‐order cortical networks (see Fig. 6), indicating that the connectivity estimates between these networks may be most affected by the nuisance regression steps. For cortical networks, within‐network connectivity estimates were generally most stable across regression options, while between‐network connections were more likely to be affected, with the exception of networks involved in higher‐order cognitive functions.

### Filtering and Autocorrelation

Another way to reduce noise and nuisance signals in resting‐state fMRI analyses is to filter the data. High‐pass filtering is typically used to remove frequencies below ∼0.008 Hz, and band‐pass filtering is typically used to additionally remove frequencies above ∼0.1Hz. Although band‐pass filtering can reduce physiological noise, it also leads to less reliable estimates of functional connectivity [Shirer et al., 2015], which may be because estimates of correlation are less efficient (more variable) when there are fewer degrees of freedom in the data. Furthermore, filtering changes the autocorrelation of a timeseries, and substantial differences in the autocorrelation between two ROIs can even bias the estimate of the correlation between them [Arbabshirani et al., 2014].

First, we investigated the levels of lag‐1 autocorrelation and its relation to age after high‐pass or band‐pass filtering. After high‐pass filtering, lag‐1 autcorrelation was higher in cortical (*r* = 0.32) than subcortical (*r* = 0.16) regions. Permutation tests demonstrated that this lag‐1 autocorrelation showed a significantly greater reduction with age in cortical (*r* = −0.37, *P* < 0.001) than subcortical (*r* = −0.15, *P* = 0.027) regions (*P* < 0.001). The differential auto‐correlation across regions that is sensitive to age may therefore bias the effects of age on estimates of functional connectivity. After band‐pass filtering, the lag‐1 autocorrelation was very high in both cortical (*r* = 0.78) and subcortical regions (*r* = 0.77), but was not correlated with age in either cortical (*r* = +0.10, *P* = 0.14) or subcortical regions (*r* = +0.04, *P* = 0.60).

Next, we compared the effects of age on connectivity estimates after band‐pass filtering (BP), high‐pass filtering (HP), and high‐pass filtering with pre‐whitening (HP‐PW). The pre‐whitening step removes auto‐correlation in the signals and may therefore lead to better estimates of functional connectivity, especially in younger adults with higher autocorrelation. We did not include the option of band‐pass filtering with pre‐whitening because we have previously found that the increased autocorrelation induced by band‐pass filtering cannot be fully removed by our standard pre‐whitening procedures [Geerligs et al., 2016]. Importantly, we prewhitened the data seperately within each ROI.

**Figure 7:**
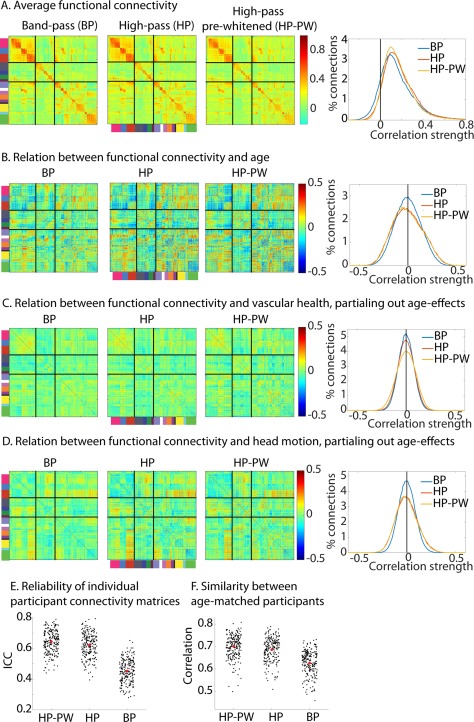
Comparison of different filtering and pre‐whitening options. (**A**) Average functional connectivity. (**B**) Relation between age and functional connectivity. The histograms show the distributions of the effects in the figures on the left. (**C**) Relation between vascular health and functional connectivity, after adjusting for effects of age. (**D**) Relation between head motion and functional connectivity, after adjusting for effects of age. (**E**) Reliability of each participants connectivity matrix (**F**) Similarity between age‐matched participants. Note all results are after regression of motion, CSF and WM signals and mean connectivity (i.e., CW + MR condition in Figure 3). BP = band‐pass filtered; HP = high‐pass filtered; HP‐PW = high‐pass filtered and pre‐whitened.

We found that the mean connectivity across participants was affected by how the data were filtered. For BP we observed stronger within network connectivity and weaker between network connectivity in higher‐order cortical networks compared to HP and PW (Fig. 7A). The pattern of age‐related changes was similar for the three types of filtering (Fig. 7B), especially between HP and HP‐PW (HP‐HP‐PW, *r* = 0.96; HP‐BP, *r* = 0.92). However, effects of age were more pronounced (i.e, had a larger range of values, both positive and negative) for HP and HP‐PW than BP (Fig. 7B).

The association between vascular health and connectivity (after accounting for age effects), on the other hand, depended only minimally on how the data were filtered. The mean correlation remained around zero (because of the regression of mean connectivity), although the spread of correlation values across all ROIs was slightly more pronounced after HP‐PW than after HP (*P* < 0.001) and more pronounced after HP than after BP (*P* < 0.001; see Fig. 7C and Table 4). The spread of correlations between head motion and connectivity (after accounting for age effects) was also significantly more pronounced after HP‐PW and HP than after BP (all *P* < 0.001; see Fig. 7D).

**Table 4:** Summary of main outcome measures for different filtering and pre‐whitening options

	HP‐PW	HP	BP
RA	0.89	0.87	0.75
RP	0.65	0.62	0.45
SP	0.700	0.686	0.626
RSW	0.415	0.404	0.278
RSB	0.352	0.332	0.200
VH	0.08	0.07	0.06
HM	0.09	0.09	0.07

*Abbreviations*: BP = band‐pass filtered; HM = Average (absolute) association between head motion and connectivity; HP = high pass filtered; HP‐PW = high pass filtered and pre‐whitened; RA = Reliability of the age‐effects; RP = Reliability of each participant's connectivity matrix; RSB = Reliability of single between‐network connections; RSW = Reliability of single within‐network connections; SP = Similarity between participants in the same age‐range; VH = Average (absolute) association between vascular health and connectivity.

The reliability of connectivity matrices of individual participants improved as the autocorrelation decreased; and was higher for HP‐PW than HP (*z* = 8.89, *P* < 0.001) and considerably higher for HP than for BP (*z* = 12.68, *P* < 0.001; see Fig. 7E and Table 4). In addition, the reliability of individual differences in connection strength was also substantially reduced with higher levels of autocorrelation, both within‐network (all *P* < 0.001) and between‐networks (all *P* < 0.001). Similarly, the reliability of the age‐effect declined with higher levels of autocorrelation (all *P* < 0.001). In addition, the similarity between age‐matched participants was higher for HP‐PW, less for HP (*z* = 8.10, *P* < 0.001) and least for BP (*z* = 12.53, *P* < 0.001; see Fig. 7F). The change in connectivity estimates between HP and HP‐PW were most substantial for those participants who had the highest amount of auto‐correlation in the HP data (*r* = 0.74, *P* < 0.001).

Next, we investigated whether these changes in reliability could be related to confounding factors. We found that the improvement in the reliability of connectivity matrices of individual participants from BP to HP was higher for participants with high levels of head motion across both sessions (*r* = +0.25, *P* < 0.001), even after adjusting for effects of age (*r* = +0.17, *P* = 0.015). No significant relation with vascular health was observed (*r* = −0.14, *P* = 0.08). In contrast, the improvement in reliability between HP and PW was not related to head motion (*r* = −0.019, *P* = 0.79) or vascular health (*r* = +0.06, *P* = 0.46).

For the similarity between age‐matched participants, we observed the same pattern, the improvement from BP to HP was higher for participants with high levels of head motion (*r* = +0.28, *P* < 0.001), even after accounting for effects of age (*r* = +0.17, *P* = 0.015). No relation to vascular health was observed (*r* = −0.02, *P* = 0.79). The improvement in the between‐participant similarity from HP to PW was related to head motion (*r* = +0.19, *P* = 0.006) and vascular health (*r* = +0.17, *P* = 0.022), however after adjusting for effects of age, the effects of head motion and vascular health were not longer significant (*r* = +0.05, *P* = 0.49; *r* = −0.05, *P* = 0.49).

These results show that part of the improvement in the reliability indices from BP to HP is due to effects of head motion; it is likely that head motion had consistent effects on connectivity estimates across scanning sessions which were reduced by removing high‐frequency signals. However, we also found that even in the 10% of participants (*n* = 21) with the lowest amount of head motion, reliability and between‐participant similarity were significantly higher for HP than BP (both *z* = 4.01, *P* < 0.001), suggesting that the improvement in reliability is also related to the lower levels of autocorrelation and increased degrees of freedom in HP compared to BP. This is confirmed by the comparison between HP and PW, where the improvement in reliability and between participant similarity does not appear to be driven by effects of head motion, but purely by the lower levels of autocorrelation and resultant decreases in autocorrelation.

### Association Between Vascular Health and Head Motion with GM and Cognitive Functioning

In the previous section, we attempted to minimize the effects of vascular health and head motion on functional connectivity estimates. One way to reduce these effects would be to directly regress out effect of head motion or effects of vascular health from our connectivity estimates across participants. Indeed, in the case of head motion, this approach has been used in a number of papers [Cao et al., 2014; Yan et al., 2013a]. However, an important concern is that individual differences in head motion and vascular health may represent a trait of participants that is associated with other individual differences, such as age‐related cognitive or neural decline [Wylie et al., 2014; Zeng et al., 2014]. A trait characteristic is consistent with the high reliability of head motion and vascular health that we observed across scans (*r* = 0.77 and *r* = 0.75, respectively). Indeed when we regressed out individual differences in vascular health or head motion from our connectivity estimates before estimating the effects of age, we observed that the reliability of the age‐effect on connectivity was reduced significantly, compared to the results with MR (mean regression: ICC = 0.89; vascular health regression: ICC = 0.80; head motion: ICC = 0.60, all *P* < 0.001). Here, we investigated whether vascular health and head motion could be related to other important dimensions of inter‐individual variability: GM volume and cognitive performance.

We used the average estimate across the two sessions as our trait measure of vascular health. We performed two VBM analyses, one for the T1 scan in each session, but we observed no significant clusters of main effects of vascular health, or interactions between vascular health and age, at a cluster corrected threshold of pFWE < 0.001. Next, we investigated the association between vascular health and cognitive functioning (as measured outside the scanner). We looked at fluid intelligence (as measured by the Cattell test), crystallized intelligence (as measured by the Spot‐the‐Word task), verbal memory (as measured by the Wechsler story recall task), mean RTs as well as RT variability (in a choice reaction time task). We found no main effects of vascular health on cognition, nor any significant interaction between vascular health and age.

**Figure 8:**
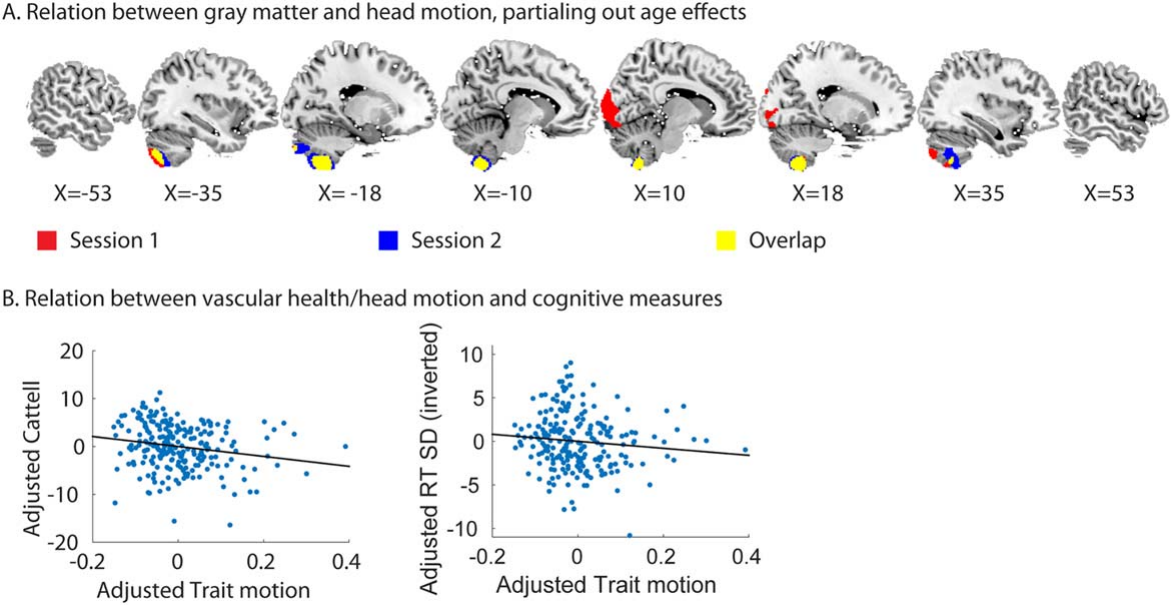
(**A**) Relation between trait motion and gray matter volume, separately for the T1 images in session 1 and session 2. (**B**) Relation between trait motion and Cattell and trait motion and response time variability. The response time variability scores were inverted, therefore higher scores indicate lower variability. Values of cognitive performance and trait motion were adjusted for effects of age.

The same analyses were performed for our measure of trait motion, which was based on the estimates of total motion from both sessions. Given that estimates of GMV can themselves be reduced by motion during a T1‐scan, we included TGM as a covariate of no interest [Reuter et al., 2015]. We performed two VBM analyses, one for the T1 scan in each session, and found that trait motion was significantly negatively‐related to GMV in the cerebellum, specifically in areas 8 and 9 (Fig. 8A) in both VBM analyses. While we cannot rule out the possibility that these VBM results are artefacts of motion during the T1 scans, their reproducibility is consistent with a biological basis of in‐scanner motion.

Next, we investigated whether in‐scanner motion related to cognitive functioning. Although there was no significant effect of motion on crystallized intelligence or verbal memory, participants with high trait motion scored lower on the Cattell test of fluid intelligence (*t*(213) = −2.49, *P* = 0.014) and had more variable RTs (*t*(213) = −2.28, *P* = 0.024) (Fig. 8B). These associations were also significant when using partial Spearman rank correlations (adjusting for effects of age), suggesting that they are not due to the effect of outliers (Cattell: *r* = −0.20, *P* = 0.002; RT SD: *r* = −0.14, *P* = 0.04) These results support the idea that individual differences in head motion have both a biological basis and a relationship with cognitive performance, in which case, regressing out motion when examining the effects of age may not be advisable as it is could remove true age‐related changes in functional connectivity, rather than simply state‐related noise.

### Functional Regions and ROI Homogeneity

Functional connectivity is typically measured by computing the Pearson correlation (Pcor) after averaging the time series of all voxels within ROIs. This may obscure true functional connectivity estimates if ROIs contain multiple sub‐regions with distinct connectivity. Previous papers have suggested that there may be an age‐related shift in the location of functional regions [Chan et al., 2014; Sohn et al., 2015], but to our knowledge this has never been tested systematically.

**Figure 9:**
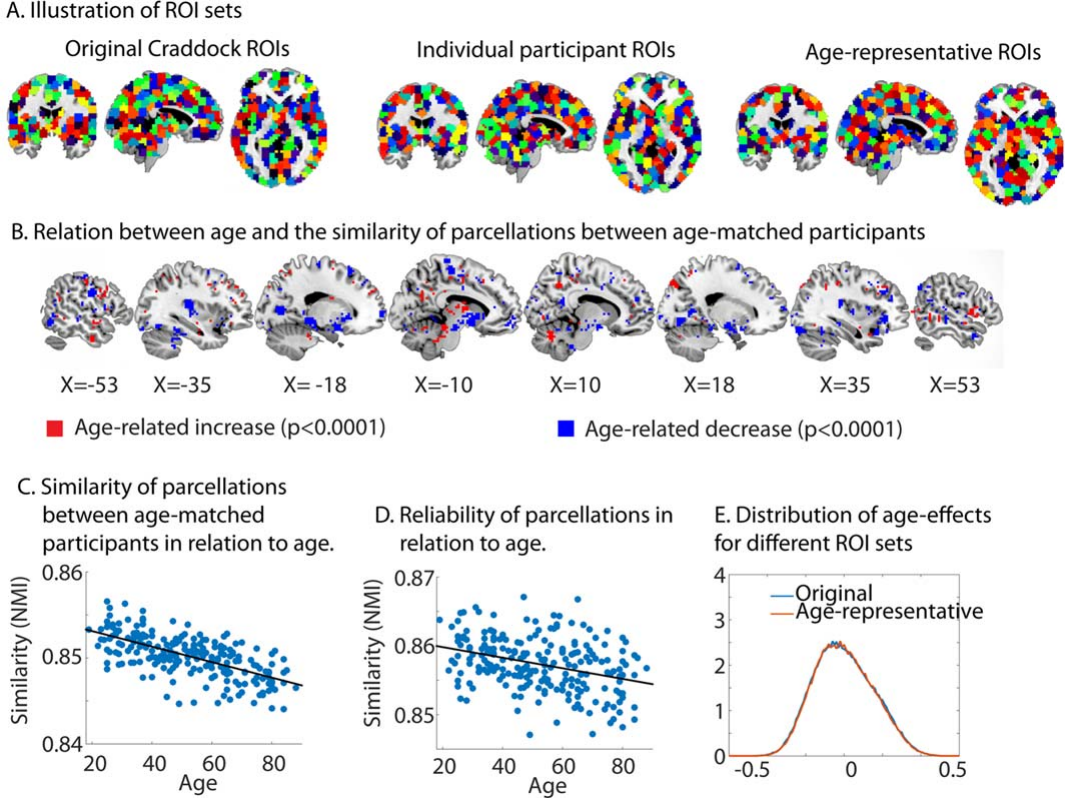
(**A**) Illustration of the different ROI sets. The individual ROIs are shown for one randomly‐selected participant. The colors of the ROIs are arbitrary. (**B**) Regions which show a change in the similarity of parcellations between age‐matched participants with age. (**C**) The relation between the similarity of parcellations between age‐matched participants and age. (**D**) Relation between the reliability of parcellations and age. (**E**) The distribution of age‐effects for the original ROI‐set and the age‐representative ROI‐set. NMI = normalized mutual information.

To investigate this, we used the methods proposed by Craddock et al. [2012] to create participant‐specific parcellations (see Methods and Fig. 9A). We then tested whether there was a consistent age‐related shift in the location of functional regions. To this end, we computed the average similarity of parcellations, using NMI between the each of the parcellations in the younger third and the oldest third of our sample. We compared this to the similarity of permuted data where the age‐labels were swapped. We found that the similarity between young and old parcellations was significantly lower than the similarity of permuted samples (*P* < 0.001), suggesting that there is a consistent age‐related shift in the location of functional regions. To investigate the localization of these effects, we constructed a binary vector for each voxel, containing ones for all the voxels in the same parcel and zeroes for voxels in another parcel. Next, we used Fisher transformed correlations as a measure of the similarity between participants for the parcellation information at each voxel. We tested for consistent age‐shifts in the location of functional regions by permuting age labels (*P* < 0.0001, 100,000 permutations). We observed that consistent age‐differences were only present in a very small number of voxels, centered on the right lateral frontal regions (BA 45), suggesting that there is not much evidence for a widespread age‐related shift in the location of functional regions.

Even though we did not find much evidence for a consistent shift in functional regions with age, there may still be age‐related changes in functional regions which vary between individuals. In this case, we would expect that the similarity between age‐matched participants would decrease with age (e.g., age related increase in idiosyncrasy). Therefore, we used NMI to measure the similarity between each participant's parcellation and that of the 30 participants that most closely matched their age. We found that this similarity with age‐peers decreased significantly with age (*r* = −0.64, *P* < 0.001, see Fig. 9C), suggesting that there is more idiosyncrasy in the location of functional regions in older adults. Similar to our analysis of consistent effects of age on functional regions, we examined the localization of these effects. Figure 9B shows the voxels in which the similarity between age‐matched participants either decreased (blue, *r* < −0.26, *P* < 0.0001) or increased (red, *r* > +0.26, *P* < 0.0001) significantly with age. We observed that the age‐related increase in idiosyncrasy reported above was mainly driven by differences in cortical regions, while some subcortical regions actually showed age‐related increases in similarity between age‐matched participants.

We examined whether this age‐related increase in idiosyncrasy could be explained by an age‐related reduction in the reliability of functional parcellations, by comparing the separate parcellations obtained from session 1 and 2. There was a reduction in the across‐scan reliability of functional parcellation in older adults (*r* = −0.34, *P* < 0.001, see Fig. 9E), but even after partialing out this reliability, the increased idiosyncrasy of functional parcellations with age remained (*r* = −0.58, *P* < 0.001).

Next, we investigated whether participant‐specific ROIs improve the homogeneity of signals within ROIs, by computing the average correlation between the time‐series of different voxels within an ROI. In the participant‐specific set, the number of ROIs varied between 696 and 746 (average 719), while the original Craddock set contained 746 ROIs. Despite the smaller number of ROIs for participant‐specific ROIs (and hence more voxels per ROI on average), we found that the similarity of voxel time‐series within an ROI improved significantly with respect to the set of original Craddock ROIs (*r* = 0.711 versus *r* = 0.708, *T*(213) = 28.1, *P* < 0.001). Homogeneity was significantly reduced with advancing age in the original ROI set (*r* = −0.135, *P* = 0.049), while this decline was not significant in the participant‐specific ROI set (*r* = −0.128, *P* = 0.063). However, when we used a parametric test to compare the strength of these correlations [Steiger, 1980], we found that the difference between these two correlation coefficients was not significant (*z* = 1.32, *P* = 0.19). We did not examine effects of participant‐specific ROIs on connectivity estimates, because it was not possible to match ROIs across participants.

These results suggest that the use of participant‐specific parcellations may lead to more valid and somewhat less age‐biased results. While participant‐specific ROIs may seem optimal, group‐based ROIs will be more accurate for those functional regions that are consistently located across participants (by virtue of pooling over more data to define them), and most importantly, a common ROI set facilitates connectivity analyses across participants. We therefore tested whether an improvement was also achieved using a group ROI set generated from our own sample, which is more age‐representative than the original Craddock sample. Indeed, this sample‐specific set of 750 ROIs showed higher homogeneity across participants than the original Craddock ROIs (0.720 versus 0.708, *T*(213) = 131.05, *P* < 0.001). This improvement was larger than for the participant‐specific ROI set, although this may owe to the larger number of ROIs (750 versus ∼719). The association between homogeneity and age was similar in the original Craddock ROIs (*r* = −0.135, *P* = 0.049) and the age‐representative ROI set (*r* = −0.136, *P* = 0.048).

The age‐representative ROIs did not reveal a change in the distribution of age‐effects compared to the original Craddock ROIs. There was also no improvement in the reliability of the individual connectivity matrices, using the age‐representative ROI set (ICC = 0.641) as compared to the original Craddock set (ICC = 0.645), nor in the reliability of individual connections (ICC = 0.356 vs. ICC = 0.357, respectively), nor in the reliability of the effects of age on functional connectivity (ICC = 0.882 vs. ICC = 0.885, for the new and the original ROIs respectively). Finally, the similarity between age‐matched participants was slightly lower for the age‐representative ROIs (*r* = 0.696), compared to the original Craddock ROIs (*r* = 0.700). These results suggest that, at least in this specific case (using the Cradock method with 750 ROIs), the use of an age‐representative, group ROI set did not appear to be advantageous.

We also examined how age affects homogeneity in a different set of ROIs, which were based on results from meta‐analyses [Power et al., 2011], instead of similarity between time‐courses. In line with the results from our previous study [Geerligs et al., 2016],we found that the homogeneity was significantly higher in the Power ROI set (*r* = 0.83) compared to the original Craddock ROIs (*r* = 0.71, *t*(214) = 178.6, *P* < 0.001). However, we also observed a greater age‐related decline in homogeneity for the Power ROI set (*r* = −0.185, *P* = 0.007), compared to the Craddock ROIs (*r* = −0.135, *P* = 0.049). The difference between these correlations was significant (*Z* = 3.24, *P* = 0.001).

**Figure 10:**
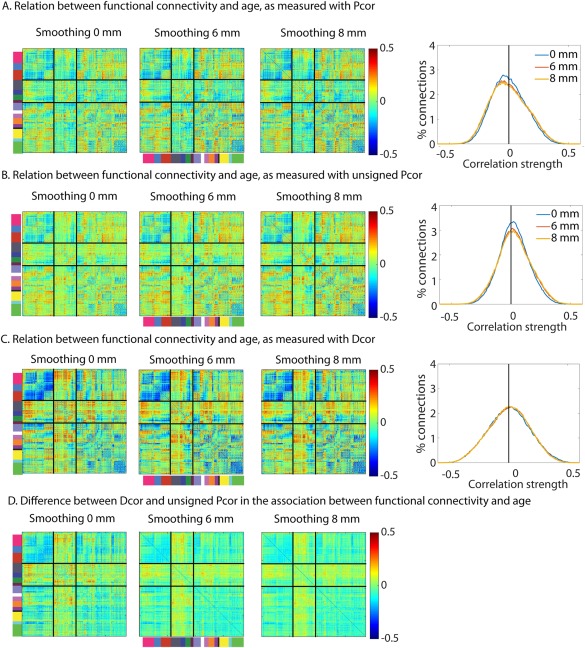
Association between age and functional connectivity for different amounts of smoothing (left to right 0 mm, 6 mm and 8 mm) and various connectivity measures (**A**) Pcor, (**B**) unsigned Pcor (**C**) Dcor. (**D**) The difference in the association between age and functional connectivity between Dcor and unsigned Pcor. Pcor = Pearson correlation; Dcor = distance correlation; abs Pcor = absolute/unsigned Pearson correlation.

**Figure 11:**
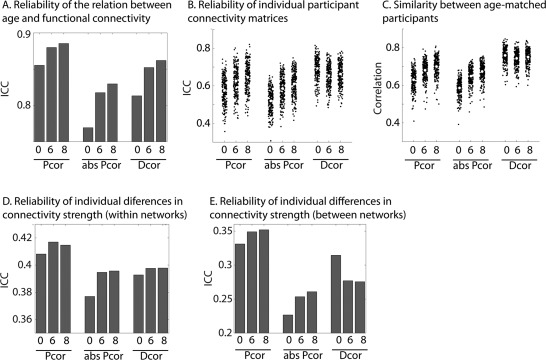
Reliability and between participant similarity for Pcor, Dcor, and unsigned (absolute value, abs) Pcor connectivity matrices with different levels of smoothing. (**A**) Reliability of the age‐connectivity matrices (**B**) Reliability of each participants connectivity matrix (**C**) Similarity between age‐matched participants (**D**) Reliability of individual differences in connectivity strength, averaged across all within network connections. (**E**) Reliability of individual differences in connectivity strength, averaged across all between network connections. Pcor = Pearson correlation; Dcor = distance correlation; abs Pcor = absolute/unsigned Pearson correlation. 0, 6, and 8 refer to the mm of smoothing that was applied to the data.

Another way to accommodate differences in the location of functional regions across participants is to smooth the data spatially (across voxels). The data in all previous analyses were smoothed with an 8 mm FWHM Gaussian kernel (S8). Here we examined how smoothing affected the homogeneity, the associations between age and connectivity and the reliability of connectivity estimates. Averages and statistical tests that are not reported in the text can be found in Tables 5 and 6 and Figure 11. We found that the age‐related decrease in ROI homogeneity got smaller as smoothing increased (S0: *r* = −0.33, *P* < 0.001; S6: *r* = −0.17, *P* = 0.011 and S8: *r* = −0.13, *P* = 0.049). When we compared these correlations we found that there was a significant increase in the association between age and homogeneity from S8, to S6 and S0 (S0 vs. S6, *z* = 5.97, *P* < 0.001; S6 vs. S8, *z* = 4.63, *P* < 0.001). The similarity of connectivity estimates between age‐matched participants also increased with smoothing. Interestingly, both the positive and the negative associations between age and functional connectivity were amplified as smoothing increased, as evidenced by a significant increase in mean absolute association between age and connectivity (Fig. 10A; S0: *M* = 0.114, *M* = 0.124; S8: *M* = 0.126; all *P* < 0.001). In addition, the age‐effects were significantly more reliable after smoothing and the reliability of individual connectivity matrices increased after smoothing. The reliability of individual differences in connectivity strength increased with smoothing for between‐network connections, while for within‐network connections an increase was observed for S6 and S8 compared to S0, but a decrease was observed for S8 compared to S6 (Fig. 11D,E, Tables 5 and 6). The increased strength of age‐effects after smoothing suggests that the misalignment of functional regions inflates the differences between participants, and thereby obscures any consistent effect of age on functional connectivity.

**Table 5:** Summary of main outcome measures for different levels of smoothing and different connectivity estimates

	Pcor			Abs Pcor			Dcor		
	S0	S6	S8	S0	S6	S8	S0	S6	S8
RA	0.86	0.88	0.89	0.77	0.82	0.83	0.81	0.85	0.86
RP	0.58	0.63	0.65	0.52	0.59	0.61	0.69	0.65	0.66
SP	0.630	0.680	0.700	0.586	0.649	0.673	0.759	0.743	0.751
RSW	0.408	0.417	0.415	0.377	0.395	0.396	0.393	0.398	0.398
RSB	0.331	0.349	0.352	0.227	0.253	0.261	0.314	0.277	0.275

*Abbreviations*: abs Pcor = absolute/unsigned Pearson correlation; Dcor = distance correlation; Pcor = Pearson correlation; RA = Reliability of the age‐effects; RP = Reliability of each participant's connectivity matrix; RSB = Reliability of single between‐network connections; RSW = Reliability of single within‐network connections; SP = Similarity between participants in the same age‐range.

S0, S6, and S8 refer to the mm of smoothing that was applied to the data.

**Table 6:** Statistical tests of main outcome measures for different levels of smoothing and different connectivity estimates

		Pcor		Dcor		S0		S6		S8	
		S6 vs. S0	S8 vs. S6	S6 vs. S0	S8 vs. S6	Dcor vs. Pcor	Dcor vs. abs Pcor	Dcor vs. Pcor	Dcor vs. abs Pcor	Dcor vs. Pcor	Dcor vs. abs Pcor
RA	Diff	0.03	0.01	0.04	0.01	−0.04	0.04	−0.03	0.04	−0.02	0.03
	Pval	<0.001	<0.001	<0.001	<0.001	<0.001	<0.001	<0.001	<0.001	<0.001	<0.001
RP	Zval	12.68	12.68	−11.84	11.54	12.65	12.68	8.55	12.66	6.47	12.59
	Pval	<0.001	<0.001	<0.001	<0.001	<0.001	<0.001	<0.001	<0.001	<0.001	<0.001
SP	Zval	12.68	12.68	−9.96	12.64	12.68	12.68	12.49	12.68	12.20	12.67
	Pval	<0.001	<0.001	<0.001	<0.001	<0.001	<0.001	<0.001	<0.001	<0.001	<0.001
RSW	Diff	0.009	−0.002	0.005	0.000	−0.015	0.016	−0.019	0.003	−0.017	0.002
	Pval	<0.001	<0.001	<0.001	0.249	<0.001	<0.001	<0.001	<0.001	<0.001	<0.001
RSB	Diff	0.018	0.003	−0.038	−0.001	−0.017	0.088	−0.072	0.024	−0.076	0.015
	Pval	<0.001	<0.001	<0.001	<0.001	<0.001	<0.001	<0.001	<0.001	<0.001	<0.001

*Abbreviations*: abs Pcor = absolute/unsigned Pearson correlation; Dcor = distance correlation; Pcor = Pearson correlation; RA = Reliability of the age‐effects; RP = Reliability of each participant's connectivity matrix; RSB = Reliability of single between‐network connections; RSW = Reliability of single within‐network connections; SP = Similarity between participants in the same age‐range.

S0, S6, and S8 refer to the mm of smoothing that was applied to the data. Diff = difference between the two estimates, this is reported for comparisons where permutation test are used. Zval = *z*‐value from the Wilcoxon signed rank test; Pval = *P*‐value.

The downside of smoothing is that it reduces the ability to localize functional connectivity to specific ROIs. Another potential solution to this problem of misalignment of functional regions that is less likely to reduce spatial specificity is to use a multivariate measure of functional connectivity. Multivariate measures utilize data from all the voxels in an ROI, instead of just the average. We have recently shown that distance correlation (Dcor) is well equipped to deal with inhomogenous ROIs [Geerligs et al., 2016]. Figure 10A,C show the association between age and functional connectivity for Dcor and Pcor. One downside of Dcor is that it is not able to distinguish between positive and negative correlations. For fairer comparability of reliability measures, we therefore also show the results for the unsigned Pcor values (Fig. 10B). We found there was a strong correspondence between the age‐related changes that were observed with unsigned Pcor and Dcor (see Fig. 10D). This correspondence increased as the amount of smoothing increased and there were fewer effects of ROI inhomogeneity on connectivity. The age‐related decreases in connectivity that were observed with Pcor, such as in the DMN, the fronto‐parietal network and the visual networks, were expressed stronger with Dcor. In addition, we observed a stronger positive effect of age for Dcor in subcortical connections, most markedly between visual and subcortical networks. This is in line with the results from our previous study, where we showed that subcortical networks have the most ROI inhomogeneity and therefore also the biggest difference in connectivity estimates between Pcor and Dcor. Furthermore, while smoothing exaggerated the pattern of age effects on connectivity for Pcor and unsigned Pcor, this was not the case for Dcor (Fig. 10A–C).

Smoothing also improved the reliability of individual connectivity matrices and single connections for both Pcor and unsigned Pcor, but not Dcor (see Fig. 11). Here, we discuss the differences between Pcor, Dcor and unsigned Pcor for data with 8 mm smoothing; results were similar across smoothing levels and results for other smoothing levels are shown in Figure 11 and Tables 5 and 6. The reliability of age‐effects with Dcor were higher than for unsigned Pcor values, although they were lower compared to the signed Pcor values. The reliability of the connectivity matrices of each individual participant were higher for Dcor than both unsigned and signed Pcor. Also, the reliability of single between‐network connections was higher for Dcor than unsigned Pcor, but lower compared to signed Pcor. Similarly, for within‐network connections, reliability was higher for Dcor compared to unsigned Pcor, but lower compared to signed Pcor. We also observed that the similarity of connectivity between age‐matched participants was higher for Dcor than signed Pcor and unsigned Pcor, and varied less with smoothing (see Fig. 11 and Tables 5 and 6). Together, these results support our previous claims that distance correlation is able to deal with misalignment between ROIs and functional regions in the brain.

### Association Between Connectivity and Cognitive Function

**Figure 12:**
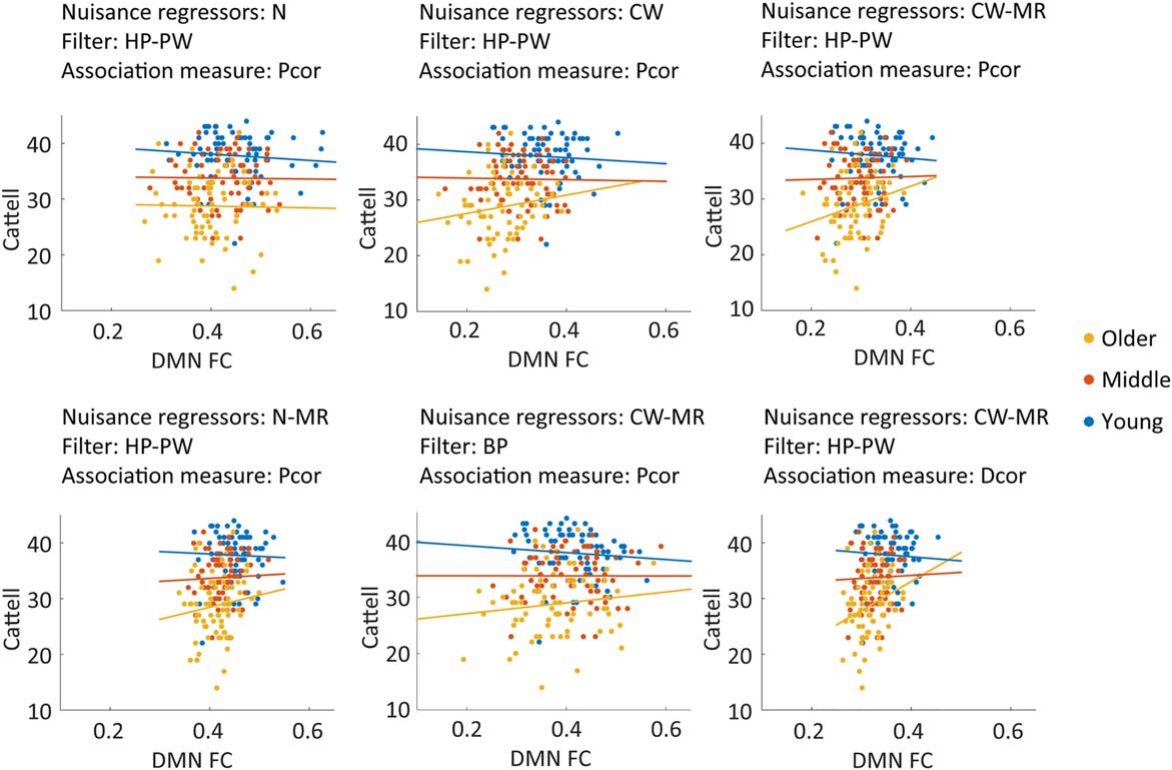
Association between DMN connectivity and performance on the Cattell task of fluid intelligence. Participants were split up into the youngest, middle and oldest third. Connectivity values were based on 8 mm smoothed data. The other nuisance regression and filtering options, as well as the choice of association measure are indicated on the top of each figure. N = only motion regressors; CW= motion regressors + CSF regressors + white matter regressors; HP = high pass filter; BP = band pass filter; PW = pre‐whitening. Pcor = Pearson correlation; Dcor = Distance correlation; MR = mean regression.

For many studies of functional connectivity, the ultimate aim is to relate connectivity to cognitive function. Given prior associations reported between the DMN and cognition [Hafkemeijer et al., 2012; Wang et al., 2013], we investigated whether the different pre‐ and post‐processing choices described above affected the association between connectivity strength within the DMN and fluid intelligence. We found that nuisance signal regression strengthened the association between connectivity and cognition: there was a significant interaction between age and DMN connectivity in relation to fluid intelligence, for CW and C, but not N. This interaction was driven by a positive association between DMN connectivity and fluid intelligence that was specific for the oldest tertile of our sample (see Fig. 12 and Table 7). This correlation between DMN connectivity and fluid intelligence in the oldest tertile was stronger (although not significant) for CW than for C and was absent in N. No main effects of DMN connectivity on fluid intelligence were observed.

**Table 7:** Results of analyses linking performance on the Cattell fluid intelligence test to DMN connectivity and age

					Interaction effect		Correlation in the older group	
Nuisance	MR	Filter	Smooth	Measure	*T*	*P*	*R*	*P*
N	N	PW	8 mm	Pcor	1.11	0.266	−0.02	0.887
CSF	N	PW	8 mm	Pcor	2.29	0.023*	0.10	0.391
WM	N	PW	8 mm	Pcor	2.34	0.020*	0.16	0.197
CC	N	PW	8 mm	Pcor	2.60	0.010*	0.15	0.215
N	Y	PW	8 mm	Pcor	2.34	0.020*	0.14	0.266
CSF	Y	PW	8 mm	Pcor	3.14	0.002*	0.27	0.024*
WM	Y	PW	8 mm	Pcor	2.79	0.006*	0.23	0.054
CC	Y	PW	8 mm	Pcor	2.21	0.028*	0.20	0.094
WM	Y	HP	8 mm	Pcor	2.29	0.023*	0.23	0.058
WM	Y	BP	8 mm	Pcor	1.80	0.073	0.13	0.277
WM	Y	PW	0 mm	Pcor	3.06	0.003*	0.27	0.028*
WM	Y	PW	0 mm	Dcor	2.30	0.022*	0.16	0.191
WM	Y	PW	8 mm	Dcor	2.97	0.003*	0.27	0.023*

*Abbreviations*: BP = band pass filter; CC = motion regressors + CompCor CSF and WM regressors; CW= motion regressors + CSF regressors + white matter regressors; Dcor = Distance correlation; HP = high pass filter; MR = mean regression; N = No mean regression; N = only motion regressors; Pcor = Pearson correlation; PW = pre‐whitening; Smooth = smoothing; Y = mean regression.

* indicates a statistically significant effect.

This interaction effects between age and DMN connectivity on Cattell scores was strengthened further using CompCor, instead of the mean CSF and WM signals. A similar increase in the interaction affect was observed after MR. However, for MR (in combination with C or CW) we additionally found that the association between DMN connectivity and fluid intelligence in the older tertile became significant. Interestingly, for the different filtering and pre‐whitening options (for CW + MR), we found that the association between DMN connectivity and Cattell was stronger for HP‐PW, weaker for HP and no longer significant for BP.

Smoothing the data slightly weakened the DMN‐age interaction. However, using multivariate Dcor increased the strength of this interaction when 8 mm smoothing was applied compared to Pcor. But this was not true when no smoothing was used.

We also investigated whether these differences between pre‐ and post‐processing choices in their association between DMN connectivity and fluid intelligence in the older group were significant. Using parametric tests [Steiger, 1980], we observed that a few of the different options showed statistically significant differences; these were the difference between no nuisance regression (N) and C‐MR (*z* = 2.08, *P* = 0.038); the difference between N and CW + MR for 0 mm smoothing (*z* = 1.97, *P* = 0.049) and the difference between N and CW + MR for 8 mm smoothing with Dcor (*z* = 2.29. *P* = 0.022). These results suggest that that pre‐ and post‐processing optimization does affect the type of outcome that is most important in many studies of functional connectivity.

## DISCUSSION

Aging is likely to have important effects on functional connectivity in the brain, but measuring connectivity with fMRI raises several challenges, such as the confounding effects of age on vascular health, head movement and functional organization. We have demonstrated that different analysis choices, designed to address some of these confounding effects, dramatically alter the effect of age observed on resting‐state fMRI connectivity. More specifically, we examined, among others, the choice of (i) nuisance signal regression and connection strength standardization, (ii) temporal filtering and pre‐whitening, (iii) group‐level motion correction, and (iv) functional parcellation (see Table 1). We discuss each of these in turn.

### Vascular Health and Brain‐Wide Increases in Mean Connectivity

Differences in functional connectivity between participants are typically (implicitly) attributed to differences in neural factors. Studies on aging are one case where vascular effects may be an important systematic confound. Tsvetanov et al. [2015] for example showed that participants with better vascular health showed higher fluctuations of activity at rest and stronger evoked responses in tasks.

Indeed, we observed that vascular health was strongly related to the functional connectivity across all ROI‐pairs. Our results demonstrate that valid comparisons between participants cannot be made when these differences in mean connectivity have not been accounted for; without accounting for differences in the mean we would conclude that aging is associated with brain‐wide decreases in neural connectivity, while these results are actually driven by physiological signals. While we have focused on effects of aging, we also observed variations in vascular health and mean connectivity in young participants (see Figs. 2 and 5G). While physical health may of course affect the true neural connectivity in the brain, our results suggested that the association with vascular health was due to the presence of brain‐wide signals, which were most likely to have a physiological, rather than neural, origin. This is in line with a recent study that has demonstrated that brain‐wide fMRI signals are associated with signals from head motion, respiration and heart rate variability [Power et al., 2017].

One way that vascular confounds might be corrected (in addition to other confounds like motion) is to regress out signals that come from voxels outside the GM ROIs, such as in WM or CSF, which are likely to contain global physiological artefacts without any neural signal [Jo et al., 2010]. We found that regression of these nuisance signals indeed reduced the effect of vascular health on connectivity and also improved the reliability of connectivity estimates across sessions. However, residual associations between vascular health and functional connectivity were observed even after regression of the mean CSF and WM signals.

Several methods can be used to reduce the effects of widespread differences in the mean connectivity. In a subset of participants, we investigated whether adding RETROICOR regressors could further reduce these effects of vascular health, but we found only minimal changes after RETROICOR, suggesting that the residual effects of vascular health (over and above variance in vascular health explained by CSF and WM) are not solely due to cardiac pulsation artifacts. These results are in line with previous findings, showing that variations in heart rate (the measure we used as a proxy for vascular health) are linked to variations in respiration [Power et al., 2017] and that both are associated with brain‐wide variations in BOLD activity [Power et al., 2017; Shmueli et al., 2007; Tsvetanov et al., 2015].

Another method that is often been applied is global signal regression, in which the mean across all brain voxels for each volume is used as a nuisance regressor within the analysis of each participant. Global signal regression shifts the distribution of connectivity values to have zero mean. Although global signal regression may reduce effects of physiological signals and head motion [Power et al., 2014; Weissenbacher et al., 2009], the global signal can also include neural signals, and the contribution of vascular and neural signals can vary across participants [Chen et al., 2012; Saad et al., 2012]. That is why we did not include global signal regression in our analysis pipeline but instead explored the ability of CompCor and MR to reduce residual effects of physiological signals on connectivity estimates. The CompCor method relies on regressing out more nuisance signals, by including not only the mean signal but the first five principal components from the CSF and WM signals [Behzadi et al., 2007; Chai et al., 2012]. MR instead works by regressing out differences in mean connectivity across participants on the group level [Yan et al., 2013b].

We found that including CompCor signals as nuisance regressors indeed reduced the effects of vascular health on functional connectivity. However, it also resulted in a strong reduction in the reliability of connectivity estimates, both across participants and across sessions. Moreover, it increased the effects of head motion on functional connectivity. This is in line with the results of previous studies: Varikuti et al. found that CompCor was disadvantageous for both within or between‐participant reliability [Varikuti et al., 2017] while Shirer et al. found a negative impact of CompCor specifically on test‐retest reliability [Shirer et al., 2015]. There may be several reasons for this decrease in reliability. First, slight changes in the nuisance signals over sessions can change the composition of the principal components and thereby result in a different set of nuisance regressors for different sessions. Second, simulation studies have shown that by regressing out too many nuisance signals, we may extract variance that is related to the underlying network structure [Bright and Murphy, 2015]. Third, there may be variability across participants in the number of components that should optimally be included [Muschelli et al., 2014] and the strongest components of the CSF and WM are not necessarily the ones that affect the ROI time series the most. Another potential explanation for the decline in reliability that is observed after CompCor is that the noise component may constitute a stable component which artificially increases the reliability of the connectivity estimates [Shirer et al., 2015]. While we cannot exclude this possibility, it is striking that other nuisance regression methods (e.g. mean CSF and WM signals and MR) led to an increase, rather than a decrease in the within, as well as between‐participant reliability indices (for the full connectivity matrices). Therefore, future research is important to further optimize the selection of nuisance components.

In contrast, MR resulted in more reliable connectivity estimates while simultaneously eliminating differences in mean connectivity between participants and reducing associations between connectivity and vascular health. It also reduced the effects of head motion on connectivity estimates; improved the reliability of the effects of age, the reliability of single connections (across participants), the reliability of the connectivity matrix (across sessions) and the similarity between participants. In addition, stronger associations between DMN connectivity and performance on the Cattell test of fluid intelligence were observed after MR. While prior to MR, the association between age and connectivity relied heavily on the choice of nuisance regressors, this was no longer the case after MR was performed. MR does make the assumption that individual differences in average levels of functional connectivity across the brain reflect physiological noise, rather than neural signal. If this assumption is not valid, the estimated age‐differences may be inaccurate. In addition, in smaller samples (where regression across participants is more susceptible to outlier values), within‐participant correction methods, such as mean subtraction [Yan et al., 2013b], may be more applicable. An alternative way to deal with overall connectivity differences would be to focus on the pattern of age‐effects, without regressing out differences in mean connectivity. For example, graphs are often binarized after equating for number of edges, thereby disregarding information about connectivity strength [Cao et al., 2014; Geerligs et al., 2015a,2015b]. However, unlike MR, these approaches would not be able to adjust for the effects of the mean in a regionally specific way.

These findings may explain some of the striking differences in the literature about the effects of age on functional connectivity. Studies using global signal regression have typically observed both increases and decreases in connectivity with age because global signal regression shifts the distribution of connectivity values toward mean zero [Andrews‐Hanna et al., 2007; Betzel et al., 2014; Chan et al., 2014; Geerligs et al., 2014, 2015a; Meier et al., 2012], whereas studies with no global signal regression, including those using independent component analysis (ICA), have shown primarily decreases in functional connectivity with age [Chou et al., 2013; Damoiseaux et al., 2008; Onoda et al., 2012], potentially due to an age‐related decrease in the contribution of physiological signals to connectivity estimates.

Many studies are interested in the association between connectivity and other characteristics of an individual, such as cognitive function, personality traits or disease symptoms. Here we have shown that the choices made in pre‐ and posts‐processing also affect the strength of the associations observed between cognition and connectivity. WM and CSF regression strengthened the association between connectivity and cognition, as did MR. In addition, we have shown that nuisance signals affected between‐network connectivity estimates most, while within‐network cortical connections were less affected. Together, these results demonstrate that pre‐ and post‐processing choices do not only affect the reliability of our results but also the conclusions that can be drawn with regards to important other variables such as cognition. Most importantly, we have demonstrated that differences between participants in mean connectivity strength need to be accounted for before valid comparisons can be made.

### Filtering, Autocorrelation, and Pre‐Whitening

Higher frequency components of the fMRI signal have been thought to reflect physiological noise, which is why many studies apply band‐pass filtering prior to calculating functional connectivity. Recently however, some studies have shown that band‐pass filtering can also impair the efficiency of functional connectivity estimates [Shirer et al., 2015] and that high‐frequency signals contain more than just physiological noise, and contribute to functional connectivity [Boubela et al., 2013; Chen and Glover, 2015], especially in subcortical areas and the insula [Kalcher et al., 2014]. One important issue is that band‐pass filtering increases the autocorrelation in the data, which in turn leads to less efficient estimation of functional connectivity [Arbabshirani et al., 2014; Geerligs et al., 2016] and potential bias if the autocorrelation differs across ROIs [Arbabshirani et al., 2014].

The current results reinforce those findings, by showing that the effects of band‐pass filtering are two sided. On the one hand, we observed that band‐passing filtering reduced the effects of vascular health and head motion on functional connectivity. However, we also found that band‐pass filtering strongly reduced the reliability of connectivity estimates and reduced the reliability of the effects age on functional connectivity. Part of this change in reliability was associated with the amount of head motion during the scan, suggesting that consistent head motion across sessions could impact functional connectivity in a consistent fashion. However, we also observed reduced reliability after band‐pass filtering in the lowest moving participants and we found that band–pass filtering weakens the association between DMN and fluid intelligence in older adults. Together, these results suggest that band‐pass filtering reduces the effects of vascular health and head motion on functional connectivity, while simultaneously impairing the efficiency of the connectivity estimation and the reliability of connectivity estimates by increasing the amount of autocorrelation and reducing the effective degrees of freedom in the data.

When the data were high‐pass filtered, we found that the dominant source of autocorrelation, at Lag 1, was stronger in younger than in older adults. This residual autocorrelation can be reduced by pre‐whitening the data. When we did this, we found that pre‐whitening (after high‐pass filtering) further improved the reliability of the age‐effect, as well as the reliability of the estimated connectivity matrices in individual participants and the similarity of connectivity estimates of age‐matched participants. These results confirm that autocorrelation is an important factor to consider when optimizing functional connectivity estimates. In some cases, high‐pass filtering combined with pre‐whitening may be a better analysis choice than band‐pass filtering, at least in studies where individual differences in head motion and other nuisance signals are not a major concern.

### Vascular Health and Head Motion: Traits and Artefacts

Head motion is another confound that is particularly relevant in the study of healthy aging, as older adult often move more than younger participants [Cao et al., 2014; D'Esposito et al., 1999; Geerligs et al., 2015a,2015b]. Even after elaborate modelling of motion parameters, differences in connectivity between high and low motion participants remain [Power et al., 2014; Satterthwaite et al., 2013; Yan et al., 2013a]. This is why some studies have regressed out total head motion across participants [Cao et al., 2014; Dai et al., 2015]. Similarly, we could reduce effects of vascular health by regressing out vascular health effects across participants. However, such group‐wide corrections reduced the reliability of the observed age‐effects. This may be because it is difficult to separate the effects of head motion/vascular health from effects of aging and aging due to their high correlation.

One might think that it is safer to examine effects of age after possible vascular health and motion artifacts have been removed from the functional connectivity estimates by group‐wide correction. However, the danger is that important effects of age on true neural connectivity are also removed. Both vascular health and head motion were stable across the two scans, suggesting a trait component above any random, state‐related noise. For vascular health, there was no evidence that this trait was related to GM volume or cognitive profile. In contrast, we found evidence that head motion has both neural and cognitive correlates. Specifically, we observed that participants who moved more had smaller GM volume in areas 8 and 9 of the cerebellum, and lower scores on tests of general cognitive decline, fluid intelligence and crystallized intelligence, as well as higher variability of RTs, even after adjusting for age. While it is difficult to disentangle true correlates of trait motion from motion artefacts, as motion during the structural scan itself may have compromised the segmentation and spatial normalization of the cerebellum, we attempted to mitigate these concerns using TGM as a covariate [Reuter et al., 2015]. Moreover, it is noteworthy that these selective regions of gray‐matter reduction not only span the secondary somatomotor representations of the cerebellum but also form part of the saliency and dorsal attention networks [Buckner et al., 2011], and so may relate to individual differences in cognitive control and working memory [Buckner, 2013]. For example, lower levels of cognitive control, as reflected by declines in fluid intelligence and RT variability [Campbell et al., 2015], may result in a participant being less able to monitor their head motion throughout the scan. Alternatively, participants with lower cognitive ability may have trouble understanding the instructions for the MRI scan, including the instruction to remain still. Motivational factors might also play a role, with participants who are less inclined to adhere to instructions to keep their head still in the scanner also being less motivated to try their best while performing various cognitive tests.

If there is a neurobiological foundation for individual differences in head motion, this may also cause differences in functional connectivity [Zeng et al., 2014]. Therefore, future studies dealing with motion artefacts in structural or functional data should be cautious about group‐level regression of average head motion, as it could lead to removal of real as well as artefactual functional connectivity differences between individuals [Wylie et al., 2014]. Moreover, group level regression may not be as effective as it seems; connectivity has been shown to change in a non‐linear fashion as the amount of head motion increases and in many studies, the effects of interest (such as age here) and effects of head motion are strongly related [Power et al., 2014]. Appropriately controlling for motion artefacts is essential to ensure the validity of findings, especially when studying individual or group differences in functional connectivity. Therefore, further optimization of denoising strategies is very important to the field.

### Age‐Related Changes in Functional Regions

Functional connectivity analyses are typically based on an a priori set of ROIs, often defined on young adults. If the location of true functional regions in the brain changes with advancing age, this approach could lead to worse overlap between functional regions and ROIs in older adults, as well as biased connectivity estimates [Sohn et al., 2015]. Here, we used clustering approaches to define participant‐specific ROIs, based on each participant's connectivity data, to examine whether there are differences in the location of functional regions with age. Although we found some evidence for a consistent effect of age on the location of functional regions, the biggest effect we observed was that older adults were more different from each other than were younger adults. This effect of age could not be explained by decreased reliability of the functional parcellations in older adults. These results suggest there is indeed a worse overlap between functional regions and ROIs with age. In line with this we also observed that the correlation of the voxel time courses within each ROI (homogeneity) decreased with age, especially for low levels of smoothing.

In this article, we specifically investigated the set of Craddock ROIs and used their methods to create participant‐specific parcellations. Other ROI sets exist that are based on different approaches/assumptions, such as identification of regions from meta‐analyses of previous task‐related studies [Power et al., 2011], or identification of borders between areas with different functional connectivity profiles [Gordon et al., 2016]. To investigate if our results are consistent across ROI sets, we also examined the homogeneity for the set of Power ROIs. There is no a priori reason to expect that age would have different effects on one parcel generation method compared to another, particularly as various methods have been found to show high levels of correspondence [Wig et al., 2013]. Indeed, we observed a similar age‐related decrease in the homogeneity of functional regions for the Power ROIs, which was more pronounced than for the Craddock ROIs. Together, these results suggest that while an age‐representative ROI template may reduce the age‐bias, it would not fully account for all the age‐related differences in the location of functional regions. Ideally, we would therefore use participant‐specific parcellations, although estimating these from the data is always prone to noise, which may out‐weigh the theoretical improvement in accuracy. Moreover, differences in the number of ROIs renders group‐level analyses more difficult. One solution to this problem is to use group‐constrained (hierarchical) parcellations [Wang et al., 2015], although this is beyond the scope of the present study.

We therefore examined two other approaches that may mitigate the age‐related change in the location of functional regions. The first is approach is simply to smooth the data across nearby voxels. This rendered the effects of age on functional connectivity more pronounced and more reliable. The disadvantage of smoothing however is that it reduces the spatial specificity of the functional connectivity results (e.g., in the extreme case of smoothing, differences between neighboring ROIs would be removed). The second approach we examined was to use a multivariate method to compute functional connectivity called distance correlation [Geerligs et al., 2016]. Because this method uses the information from all the voxels in an ROI, it is less affected by the low ROI homogeneity that is expected to occur with a misalignment between ROIs and true functional regions. In support of this, we observed that the similarity between participants did not increase with smoothing when distance correlation was used, and the strength of the age‐effect did not vary as much with the levels of smoothness. Interestingly, the associations between age and functional connectivity between subcortical regions were stronger for distance correlation compared to Pearson correlation. This is also in line with our previous study, where we observed that ROIs in subcortical regions tend to have the lower homogeneity than cortical ROIs [Geerligs et al., 2016]. These results suggest that multivariate methods such as distance correlation may be especially suitable for the study of aging, as they are better able to deal with the inhomogeneity that occurs due to misalignment between functional regions and ROIs. However, it should be noted that distance correlation can be biased when there is autocorrelation in the signal; therefore, it should only be used in combination with high‐pass filtering and pre‐whitening (not band‐pass filtering) [Geerligs et al., 2016].

### Limitations and Future Directions

Our measure of vascular health was based on heart rate variability, derived from ECG data during a separate MEG scan. Age‐related reductions in heart rate variability have previously been linked to changes in the regulation of the autonomous nervous system, and low frequency oscillations in the heart rate have been shown to contribute around 20% to the cerebral hemodynamics [Katura et al., 2006; Reardon and Malik, 1996; Zulfiqar et al., 2010]. So although it is likely that our measure of vascular health was related to individual differences in cerebral blood flow, it is possible that the associations between functional connectivity and vascular health we observed were related to individual differences in cardiac or general health, which did not directly affect cerebral blood flow. Future research using more direct measures of cerebral blood flow, such as from arterial spin labelling, is needed to further substantiate these findings.

In this article, we have focused on a range of methods to optimize the validity of connectivity estimates. One category of de‐noising methods that we did not consider are those based on independent component analysis (ICA). These methods, such as FMRIB's ICA‐based X‐noiseifier (FIX) and ICA‐based strategy for Automatic Removal of Motion Artifacts (ICA‐AROMA), separate the data of each individual into multiple independent components and use either classifiers (FIX) or matching with template features (ICA‐AROMA) to segregate signal from noise components [Pruim et al., 2015; Salimi‐Khorshidi et al., 2014]. While FIX is a generic noise removal algorithm, it requires retraining of the classifier for new datasets. ICA‐AROMA does not require training but can only remove head motion related noise components. Another recent advance in obtaining valid estimates of functional connectivity is the use of multi‐echo fMRI data in combination with ICA to distinguish components of the signal that are due to the BOLD response (by virtue of having a relationship between echo‐time) from components that are associated with head motion or scanner drift (which do not relate to echo‐time) [Kundu et al., 2012]. These methods are promising new directions, particularly for removing effects of head motion. Note that with our current pre‐processing strategies, we observed significant residual effects of head motion, which were more pronounced than effects of vascular health after MR. In addition, it is possible that we have underestimated true head motion effects due to the strong correlation between age and head motion in our sample (we reported the effects of head motion after removing effects of age). ICA de‐noising methods may be better equipped to account for effects of head motion, although these methods are not able to account for individual differences in global physiological signals in relation to vascular health because they rely on identifying spatially independent components [Power et al., 2017].

We focused specifically on correlation‐based measures of connectivity. It is not obvious how all of these recommendations translate to other approaches, such as group‐level spatial ICA analyses. The effects of smoothing we reported here would be expected to be comparable across correlation‐based and group‐level ICA‐based methods, as the misalignment of functional regions does not depend on the connectivity method that is used. For both types of analyses, we would expect to see reduced effects of age with lower levels of smoothing. It is less clear how the effects of physiological signals we reported here would translate to group ICA analyses. In contrast to correlation‐based analyses, studies using ICA typically do not regress out nuisance signals before the analysis. Instead, the assumption is made that the ICA will separate signal and noise components. Spatial ICA separates the data into spatially‐independent components. Because this independence is forced, it does not detect components related to brain‐wide physiological signals, such as those related to vascular health. This is unlike temporal‐ICA where such brain‐wide signals can be detected [Smith et al., 2012]. It is unclear how this forced independence between components would affect the results for participants with differing amounts of brain‐wide physiological signals. While it has been shown that global signal regression (which forces the removal of brain‐wide shared signals across voxels) can bias observed connectivity differences between participants [Saad et al., 2012], it is not clear how independent component analysis would be affected. This may also depend on how the data is centered before the analysis is performed. In addition, physiological signals that are more regionally specific will only result in a separate “noise components” if these signals have the same spatial pattern across participants; when they do not, nuisance signals and neural signals will be mixed. Although some work has directly compared results of ICA and correlation‐based connectivity estimates [Joel et al., 2011], data on how these different methods affect group differences and how they differ in effects of nuisance signals are still lacking.

### Recommendations and Conclusion

Our results demonstrate that different pre‐processing choices can substantially alter the effects that age has on fMRI resting‐state functional connectivity. First, the effects of age on the distribution of connectivity values are strongly modulated by the choice of nuisance regressors, suggesting that changes in mean connectivity may not be very meaningful. Second, the connectivity values were strongly related to vascular health, which decreases with age. This effect of vascular health can be reduced by including nuisance regressors that capture the physiological signals present in WM and CSF voxels. In addition, we propose that it is more appropriate to focus on the relative pattern of age‐related changes across ROIs; MR increases the interpretability of comparisons between participants and leads to more reliable connectivity estimates that are less affected by head motion and vascular health. We also recommend researchers to consider in each study whether band‐pass filtering is required, because it is likely to decrease the reliability of connectivity estimates. Regarding effects of head motion, we would suggest that researchers attempt to optimally reduce motion effects on the single subject level, as group level regression may not account for all head motion effects [Power et al., 2014] and because head motion appears to be a trait of participants that is associated with changes in brain structure and poorer cognition. Finally, the location of functional regions is more variable in older adults, which may lead to biased results when connectivity estimates are based on the average signal from each ROI. In the absence of reliable participant‐specific ROIs, we suggest this problem can be addressed using multivariate techniques such as distance correlation, although if one is also interested in the sign (direction) of connectivity, then we suggest smoothing the data to improve robustness of the conventional univariate Pearson correlation. These proposed choices are summarized in last two rows of Table 1.

Although this article is specifically about aging, most studies comparing different populations (e.g., patients and controls), and even studies in healthy younger populations, will be affected by similar issues. Therefore, it is imperative that future studies consider carefully the various analysis choices they make, and ideally ensure that their conclusions hold across a range of such choices.
